# Fundamental rate-loss trade-off for the quantum internet

**DOI:** 10.1038/ncomms13523

**Published:** 2016-11-25

**Authors:** Koji Azuma, Akihiro Mizutani, Hoi-Kwong Lo

**Affiliations:** 1 NTT Basic Research Laboratories, NTT Corporation, 3-1 Morinosato Wakamiya, Atsugi, Kanagawa 243-0198, Japan; 2 Department of Materials Engineering Science, Graduate School of Engineering Science, Osaka University, 1-3 Machikaneyama, Toyonaka, Osaka 560-8531, Japan; 3 Center for Quantum Information and Quantum Control (CQIQC), University of Toronto, Toronto, Ontario M5S 3G4, Canada; 4 Department of Physics, University of Toronto, 60 St. George St., Toronto, Ontario M5S 1A7, Canada; 5 The Edward S. Rogers Sr. Department of Electrical & Computer Engineering, University of Toronto, 10 King's College Road, Toronto, Ontario M5S 3G4, Canada

## Abstract

The quantum internet holds promise for achieving quantum communication—such as quantum teleportation and quantum key distribution (QKD)—freely between any clients all over the globe, as well as for the simulation of the evolution of quantum many-body systems. The most primitive function of the quantum internet is to provide quantum entanglement or a secret key to two points efficiently, by using intermediate nodes connected by optical channels with each other. Here we derive a fundamental rate-loss trade-off for a quantum internet protocol, by generalizing the Takeoka–Guha–Wilde bound to be applicable to any network topology. This trade-off has essentially no scaling gap with the quantum communication efficiencies of protocols known to be indispensable to long-distance quantum communication, such as intercity QKD and quantum repeaters. Our result—putting a practical but general limitation on the quantum internet—enables us to grasp the potential of the future quantum internet.

## Abstract (web summary)

In a future quantum internet, entanglement or a secret key should be efficiently provided between two points via intermediate nodes connected by optical channels. Here the authors derive general rate-loss trade-off for such protocols, that is applicable to any network topology.

## Graphical abstract

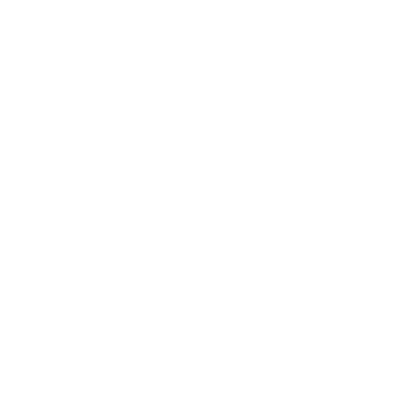

In the conventional Internet, if a client, Alice, wants to communicate with another client, Bob, an Internet protocol determines the path that the data follow to travel across multiple networks from Alice to Bob. Analogously, in the future, according to a request for performing quantum communication between Alice and Bob, a quantum internet^1^ protocol will supply the resources—such as a secret key (secret bits) for the purpose of the unconditionally secure communication^2^,^3^ and quantum entanglement (ebits) for the purpose of the quantum teleportation^4^—to Alice and Bob by utilizing proper intermediate nodes connected by optical channels—for instance, optical fibres—with each other^1^ (Fig. 1a). To such an optical network, photon loss in the optical channels is the dominant impediment in general^5^. Nonetheless, as long as Alice and Bob are not too far away from each other, say over a couple of hundred kilometres, the intermediate nodes would not be necessary, because the current point-to-point quantum communication has already been very efficient as well as ready for practical use^6^. Besides, in terms of the communication efficiency for the distance, known optical schemes^2^,^7^,^8^,^9^,^10^,^11^,^12^,^13^ for the point-to-point links are shown to have no scaling gap with an upper bound on the quantum capacity and the private capacity of the lossy optical channel, called Takeoka–Guha–Wilde (TGW) bound^14^,^15^.

In general, the TGW bound can be estimated and applied to any secret key or entanglement distillation scheme by two parties who are allowed to use their given arbitrary quantum channel(s) as well as arbitrary local operations and arbitrary classical communication (LOCC). In fact, by using this feature, the TGW bound is used to upper bound the quantum capacity and the private capacity of the lossy optical channel. This is notable because it is intractable to estimate the quantum capacity and the private capacity in general, owing to possibly non-additive nature^16^ of quantum channels. On the other hand, Pirandola, Laurenza, Ottaviani and Banchi (PLOB) have succeeded^17^ in determining the quantum capacity and the private capacity of the lossy optical channel, via finding out the teleportation stretchability of the lossy optical channel and deriving an upper bound—called PLOB bound—applied to any teleportation stretchable quantum channel. In terms of the communication efficiency described by obtained ebits or secret bits per used optical mode, the PLOB bound is, at most, twice as tight as the TGW bound for lossy optical channels. But it is still an open question which of these bounds is tighter for general quantum channels. The TGW bound applies to arbitrary quantum channels, while the PLOB bound applies only to teleportation stretchable quantum channels (although including many practical bosonic channels^17^).

Despite the differences in advantage and disadvantage between the TGW bound and the PLOB bound, perhaps most importantly in practice, both of them show that there remains not much room to improve known optical quantum communication schemes^2^,^7^,^8^,^9^,^10^,^11^,^12^,^13^ for point-to-point links further. Unfortunately, the point-to-point communication is not efficient enough to achieve the quantum internet. For example, the point-to-point quantum communication over 1,000 km needs^18^ to take almost one century to provide just one secret bit or one ebit for Alice and Bob under the use of a typical standard telecom optical fibre with loss of about 0.2 dB km^−1^. Therefore, for the request from far distant Alice and Bob, the quantum internet necessitates long-distance quantum communication schemes utilizing intermediate nodes, such as intercity quantum key distribution (QKD) protocols^19^,^20^,^21^ and quantum repeaters^18^,^22^,^23^,^24^,^25^,^26^,^27^,^28^,^29^,^30^,^31^,^32^,^33^,^34^,^35^,^36^. In particular, these schemes would be in greater demand for the quantum internet than the point-to-point quantum communication, analogously to the current Internet, where a client communicates with a far distant client via repeater nodes routinely and even unconsciously. Therefore, it is important to go beyond upper bounds (such as the TGW bound and the PLOB bound) for point-to-point links and work out fundamental and general upper bounds for a quantum internet. *A priori* working out bounds on secure key rates and entanglement generation rates for a general quantum internet topology is highly non-trivial because there are many intermediate nodes, various elements such as quantum memories and optical devices and many different protocols such as entanglement generation, entanglement swapping, entanglement distillation and quantum error correction. For this reason, up till now, a good fundamental and general upper bound on secure key rates and entanglement generation rates for the quantum internet has been missing.

The main point of this paper is to present a fundamental and practical limitation on the quantum internet. In particular, we derive rate-loss trade-offs for any two-party quantum communication over the quantum internet—composed of the use of optical fibres connecting nodes as well as arbitrary LOCC, by tailoring the TGW bound to being applicable to any network topology. The key insight is reduction. Given any quantum network (which might be a subnetwork of a quantum internet), Alice's node *A* and Bob's node *B*, we can consider any bipartition of the nodes in the quantum network, *V*_*A*_ including node *A* and *V*_*B*_ containing node *B* (cf. Fig. 1a). By regarding all nodes in *V*_*A*_ as local at *A* and all nodes in *V*_*B*_ as local at *B*—which could never increase the difficulty of quantum communication between *A* and *B*, one could reduce any network flow as a flow over a point-to-point link between *A* and *B* only. Therefore, an upper bound on the key rate or the entanglement generation rate for the point-to-point links automatically carries over to an upper bound to the quantum network. As this upper bound for point-to-point links, we simply use the TGW bound with respecting its generality, in contrast to Pirandola's contemporary work^37^, which instead uses the PLOB bound to obtain a good bound for multipath networks composed of lossy optical channels. Our reduction idea is a simple observation. Nonetheless, rather remarkably, we will show here that the obtained bounds are excellent in the sense that they have no scaling gap with achievable quantum communication efficiencies of known protocols for intercity QKD and quantum repeaters, in terms of rate-loss trade-offs. Moreover, thanks to inheriting the generality of the TGW bound, in contrast to Pirandola's bounds^37^ applied only to teleportation stretchable quantum channel networks, our bounds can be estimated and applied to any situation that can be regarded as the quantum internet as Kimble has considered^1^, including the simulation of the quantum many-body systems as well as purely quantum communication tasks. As a non-trivial example to imply this, we present upper bounds on the performance of any Duan–Lukin–Cirac–Zoller (DLCZ)-type quantum repeater protocol^18^,^23^,^24^,^30^ by considering not only loss of optical channels but also time-dependent decay of matter quantum memories. These bounds conclude that the coherence time of the matter quantum memories should be, at least, larger than 100 μs for enjoying the blessing of the DLCZ-type quantum repeaters even if they are equipped with any single-shot quantum error correction, as well as any entanglement distillation. The key to obtain these results is the fact that our bounds essentially depend only on the number of the channel uses to establish a quantum communication resource for Alice and Bob and the squashed entanglement^14^,^15^ of the used quantum channels—which is a single-letter formula that can be evaluated as a function of a single-channel use.

## Results

### Quantum internet protocol for two clients

**Figure 1:**
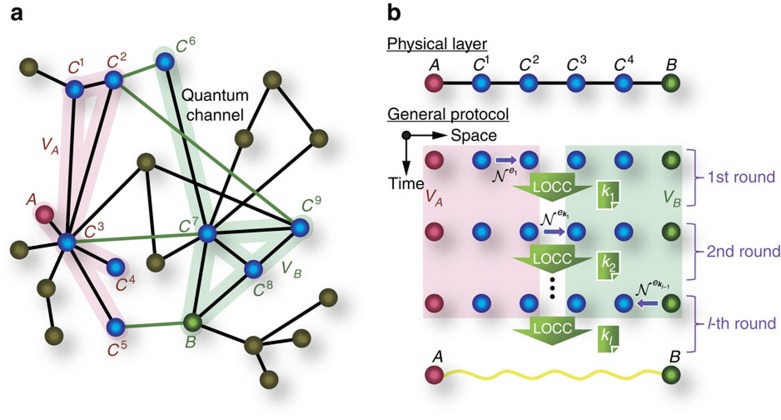
Quantum internet and the most general protocol. (**a**) A general quantum internet where Alice (*A*) and Bob (*B*) request its internet protocol to supply them with resources for quantum communication, such as a secret-key and quantum entanglement. Accordingly, the protocol chooses a quantum network *G* (which might be a quantum subnetwork) associated with a directed graph *G*=(*V*, *E*). The set *V* of vertices is composed of the nodes as *V*={*A*, *B*, *C*^1^, *C*^2^, …, *C*^*n*^} (*n*=9 here) and the set *E* of edges specifies quantum channels 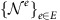 in such a way that 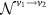 represents a quantum channel to send a quantum system from node *v*_1_∈*V* to node *v*_2_∈*V*. The protocol can combine the channels 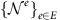 with LOCC arbitrarily. Then, we regard any protocol as the point-to-point communication between a single parity having nodes *V*_*A*_⊂*V* with *A* and another party having *V*_*B*_(=*V*\*V*_*A*_) with *B*. As a result, we obtain equation (1) showing that average obtainable ebits or secret bits are approximately upper bounded by the average of the squashed entanglement of used quantum channels between *V*_*A*_ and *V*_*B*_. In **b**, we describe the most general protocol, by exemplifying a linear network with *n*=4. The protocol starts by preparing a separable state and then by using a quantum channel 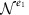. In the *i*-th round (*i*=1, 2, …, *l*), according to the previous outcomes ***k***_*i*−1_=*k*_*i*−1_ … *k*_2_*k*_1_ (***k***_0_:=1), the protocol may use a quantum channel 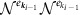 with 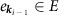, followed by LOCC providing a quantum state 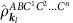 with a new outcome *k*_*i*_. After an *l*-th round, Alice and Bob obtain a quantum state 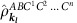, from which they can distil 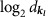 ebits or secret bits approximately.

To obtain our bound, we need to define a general paradigm of two-party communication over the quantum internet (Fig. 1a). In the quantum internet, there are a variety of quantum channels connecting nodes, for example, depending on the lengths of optical channels. This necessitates to generalize the paradigm^14^,^15^ of Takeoka *et al*. for the point-to-point communication, where it has been enough to treat only one optical channel between Alice and Bob. For instance, we need to allow the choice of which channel to use in the next round to depend on the outcomes of LOCC operations in previous rounds, in contrast to the paradigm of Takeoka *et al*.^14^,^15^.

To make this more precise, let us define the most general protocol. We assume that any classical communication over the network is freely usable. Suppose that Alice (*A*) and Bob (*B*) call a quantum internet protocol to share a resource for quantum communication, a secret key or quantum entanglement, over the quantum network. Accordingly, the quantum internet protocol determines a subnetwork to supply the resource to Alice and Bob. The subnetwork is characterized by a directed graph *G*=(*V*, *E*) with a set *V* of vertices and a set *E* of edges, where the vertices of *G* represent Alice's node, Bob's node and intermediate nodes {*C^j^*}_*j*__=1,2,…,*n*_ in the subnetwork, that is, *V*={*A*, *B*, *C*^1^, *C*^2^, …, *C*^*n*^}, and an edge 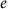
=*v*_1_→*v*_2_∈*E* of *G* for *v*_1_, *v*_2_∈*V* specifies a quantum channel 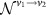 to send a quantum system from node *v*_1_ to node *v*_2_ in the subnetwork. Then, the most general protocol proceeds in an adaptive manner as follows (cf. Fig. 1b, which exemplifies a linear network with *n*=4). The protocol starts by preparing the whole system in a separable state 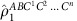 and then by using a quantum channel 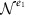 with *e*_1_∈*E*. This is followed by arbitrary LOCC among all the nodes, which gives an outcome *k*_1_ and a quantum state 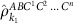 with probability 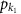. In the second round, depending on the outcome *k*_1_, a node may use a quantum channel 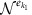 with 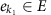, followed by LOCC among all the nodes. This LOCC gives an outcome *k*_2_ and a quantum state 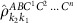 with probability 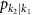. Similarly, in the *i*-th round, according to the previous outcomes ***k***_*i*−1_:=*k*_*i*−1_ … *k*_2_*k*_1_ (with ***k***_0_:=1), the protocol may use a quantum channel 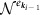 with 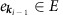, followed by LOCC providing a quantum state 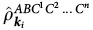 with a new outcome *k*_*i*_ with probability 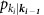. After a number of rounds, say after an *l*-th round, the protocol must present 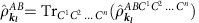 close to a target state 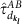 in the sense of 
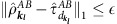
 for 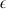
>0, from which Alice and Bob can distil 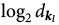 secret bits or 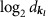 ebits. After all, the protocol results in presenting 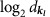 secret bits or ebits with probability 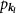 by using quantum channels 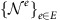, where 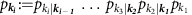.

### Fundamental limitation on the quantum internet protocol

For the general adaptive quantum internet protocol, our main result is described as follows. Let us divide set *V* into two disjoint sets, *V*_*A*_ including *A* and *V*_*B*_ including *B*, satisfying *V*_*A*_=*V*\*V*_*B*_ (and *V*_*B*_=*V*\*V*_*A*_) (see Fig. 1 for the examples). For given ***k***_*i*_, if the protocol uses a quantum channel 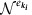 between a node in *V*_*A*_ and a node in *V*_*B*_, we write ***k***_*i*_∈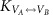. For example, ***k***_1_∈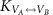 in Fig. 1b. Then, for any choice of *V*_*A*_ (or *V*_*B*_), the most general protocol has a limitation described by


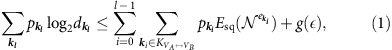


where *g* is a continuous function^14^,^38^ with the property of 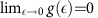 and 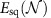 is the squashed entanglement^14^,^15^ of channel 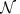. This bound is reduced to 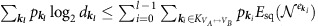 for 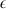
→0. The bound (1) is obtained by regarding the general protocol as bipartite communication between *V*_*A*_ and *V*_*B*_ and by applying the TGW bound to the bipartite one (see Supplementary Note 1 for the proof). Since the bound holds for any choice of *V*_*A*_, the bound shows that the average of the obtained secret bits or ebits is most tightly bounded by the choice of *V*_*A*_ minimizing the right-hand side of equation (1). Again, note that our bound (1) is applicable to any quantum network composed of arbitrary quantum channels, in contrast to Pirandola's one^37^ with the assumption of the teleportation stretchability for quantum channels.

### Application to general linear networks

As an instructive application of the bound (1), we first derive an upper bound for a general linear network as in Fig. 1b, which includes intercity QKD protocols and quantum repeater protocols as special cases. Here the goal of Alice and Bob is to share secret bits or ebits by using a quantum internet protocol with help of intermediate nodes {*C^j^*}_*j*__=1,2,…,*n*_. Suppose that the nodes *A*, *C*^1^, *C*^2^, …, *C*^*n*^ and *B* line in order (Fig. 1b), and nearest-neighbouring nodes are connected by quantum channels 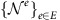, respectively. For clarity, if an edge *e* associated with a quantum channel 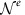 is *v*_1_→*v*_2_ or *v*_2_→*v*_1_ for *v*_1_, *v*_2_∈*V*, we refer to the edge as *v*_1_↔*v*_2_. Nodes *A* and *B* are dubbed *C*^0^ and *C*^*n*+1^, respectively (that is, *A*=:*C*^0^ and *B*=:*C*^*n*+1^). Then, as shown in Methods, from equation (1), we obtain a bound for the protocol


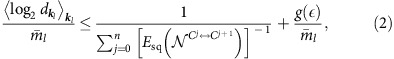


where 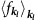 represents the average of function 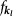 over ***k***_*l*_ and 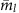 is the average total number of channel uses. The first term of the right-hand side in this inequality is proportional to the harmonic mean of the squashed entanglement of channels 
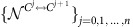
. Also, note that the left-hand side quantity—which is the average obtained secret bits or ebits per average total channel use—is different from Pirandola's measure^37^ for the performance (see Methods).

### Optimal scaling for intercity QKD and quantum repeaters

To show how good the bound (2) is, let us start by comparing it with the performance of intercity QKD protocols and quantum repeater protocols. For simplicity, suppose that all the nodes {*C*^*j*^}_*j*__=0,1,…,*n*+1_ are located at regular intervals between Alice and Bob separated over distance *L* and they are connected with optical fibres with transmittance 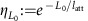 for attenuation length *l*_att_ and *L*_0_:=*L*/(*n*+1) with each other. Then, all the channels 
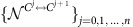
 must be the same lossy optical channel 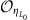 with transmittance 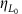, for which Takeoka *et al*.^14^,^15^ have already derived an upper bound on the squashed entanglement of the channel. This implies 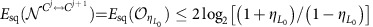 for any *j*=0, 1, …, *n*, where the factor 2 in the front comes from the fact that a single use of an optical channel for transmission of an optical pulse corresponds to the sending of two optical modes associated with its polarization degrees of freedom. Then, the bound (2) is reduced to


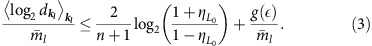


In particular, this bound shows that the average secret bits or ebits per average total channel use, 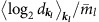, are upper bounded by 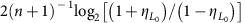 for 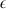
→0, which is approximated to be 4[(*n*+1)ln2]^−1^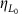 for *L*_0_≫1. The bound (3) is strong enough to show that the existing intercity QKD protocols and quantum repeater protocols are pretty good in the sense that they have the same scaling with this simple bound.

**Figure 2:**
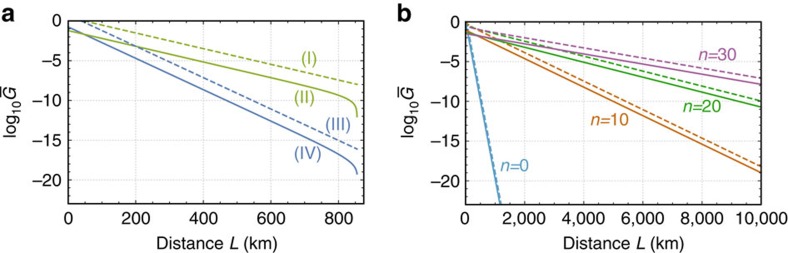
Performance and upper bounds of protocols based on linear lossy optical channel networks. The performance is measured in terms of secret bits or ebits per average total channel use, 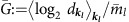, for the distance *L* between Alice and Bob. As in Fig. 1b, the protocols use intermediate nodes {*C*^1^, *C*^2^, …, *C*^*n*^} connected by optical fibres with transmittance 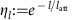 for attenuation length *l*_att_=22 km with each other and located at regular intervals, say *L*_0_=*L*/(*n*+1). The solid curves represent achievable performance, while the dashed curves are the upper bounds in equation (3) for the linear network, for various *n*. In **a**, we provide the performance of mdiQKD protocols^21^,^39^ using only a single intermediate node (*n*=1) equipped with feasible optical devices. In particular, lines (II) and (IV) represent the all-photonic intercity QKD protocol^21^ and the original mdiQKD protocol^39^, respectively. These lines just refer to the performance given in Fig. 3 of ref. 21 (see ref. 21 for the detail of the assumed optical devices). The key rate scales linearly with *η*_*L*_ for the mdiQKD^39^, but it scales linearly with *η*_*L*/2_ for the all-photonic intercity QKD^21^. We also show our bound (3) for *n*=1 as line (I) and the TGW bound^14^ (corresponding to our bound with *n*=0) as line (III). Comparing lines (I) and (II), we can see that the all-photonic intercity QKD protocol has the same scaling with our bound (3) for *n*=1. In **b**, for various *n*, we provide the performance of the idealized qubit-based quantum repeater protocol, 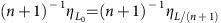, as solid lines and our bound (3) as dashed curves. We can see that there is essentially no scaling gap between our bound (3) and the idealized qubit-based protocol.

Let us first compare the bound (3) with the intercity QKD protocols^19^,^20^,^21^. This class of QKD protocols leads to a square root improvement in the secret key rate over conventional QKD schemes (without quantum repeaters) bounded by the TGW bound. Nonetheless, it is implementable^21^ without the need of matter quantum memories or quantum error correction, which is in a striking contrast to quantum repeaters^18^,^22^,^23^,^24^,^25^,^26^,^27^,^28^,^29^,^30^,^31^,^32^,^33^,^34^,^35^,^36^. In particular, those intercity QKD protocols are modifications of the measurement-device-independent QKD^39^ (mdiQKD), and all of them use a single (untrusted) intermediate node *C* in the middle of communicators Alice and Bob. Node *C* shares optical channels with Alice and Bob, whose transmittance is described by *η*_*L*/2_. By using these channels, Alice and Bob send single photons to the node *C*. Then, using matter quantum memories^19^,^20^ or using optical devices alone^21^, the middle node *C* performs the Bell measurement only on pairs of photons that have successfully survived the loss during the transmission from Alice and Bob. Since the success of the Bell measurement provides secret bits, the average secret bits of these protocols per total channel use are in the order of the survival probability of photons, that is, *η*_*L*/2_. However, this is exactly the same scaling of the bound (3), because the bound (3) is proportional to 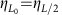 for *n*=1, 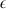
→0 and *L*_0_≫1. In fact, this is easily confirmed by seeing Fig. 2a. Therefore, it is concluded that the intercity QKD protocols^19^,^20^,^21^ have no scaling gap with the upper bound (3).

Next, let us compare the bound (3) with the performance of achievable quantum repeater protocols. Actually, there are many quantum repeater schemes^18^,^22^,^23^,^24^,^25^,^26^,^27^,^28^,^29^,^30^,^31^,^32^,^33^,^34^,^35^,^36^, depending on the assumed devices of the repeater nodes {*C*^1^, *C*^2^, …, *C*^*n*^}. For instance, a protocol assumes repeater nodes equipped with atomic-ensemble quantum memories as well as optical devices^18^,^23^. To obtain better scaling, instead of the atomic-ensemble quantum memories, another protocols^22^,^27^,^28^,^29^,^32^,^34^ use matter qubits satisfying^35^,^36^ all the criteria given by DiVincenzo^40^. Moreover, there is even an all-photonic scheme^35^ that does not use matter quantum memories at all and works by using only optical devices. However, since our aim here is to show the existence of a quantum repeater protocol that has the same scaling with the bound (3) in principle, let us introduce an idealized qubit-based protocol that uses a noiseless quantum computer with the function of the perfect coupling with single photons at each repeater node. This protocol is conceptually simple. But it gives a good lower bound of the secure key rate or the entanglement generation rate in the sense that it has the same scaling behaviour as the bound (3).

In the idealized qubit-based protocol, (i) node *C*^*j*^ (*j*=0, 1, …, *n*) begins by producing a single photon, which is in maximally entangled state |Φ^+^〉=(|0〉|*H*〉+|1〉|*V*〉)/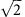 with a qubit of a local quantum computer, where {|*H*〉, |*V*〉} is an orthonormal basis for the polarization degrees of freedom of the single photon and {|0〉, |1〉} is a computational basis of the qubit. (ii) Then, the node *C*^*j*^ sends its right-hand-side adjacent node *C*^*j*+1^ the single photon through the optical fibre with transmittance 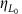. (iii) On receiving the photon from the node *C*^*j*^, the node *C*^*j*+1^ performs a quantum non-demolition measurement to confirm the successful arrival of the single photon, and announces the measurement outcome to node *C*^*j*^ via a heralding signal. If this quantum non-demolition measurement proves the successful arrival of the single photon, the node *C*^*j*+1^ transfers the quantum state of the received photon into a qubit of the local quantum computer faithfully, establishing a maximally entangled state between quantum computers in the node *C*^*j*^ and in the node *C*^*j*+1^. (iv) If the node *C*^*j*^ is informed of the loss of the sent photon in the transmission by the heralding signal from the right-hand-side adjacent node *C*^*j*+1^, the nodes *C*^*j*^ and *C*^*j*+1^ repeat steps (i)–(iii). (v) If every node shares a maximally entangled state with the adjacent nodes, all the repeater nodes {*C*^1^, *C*^2^, …, *C*^*n*^} apply the Bell measurement to a pair of local qubits that have been entangled with qubits in the adjacent repeater nodes. This gives Alice and Bob a pair of qubits in a maximally entangled state.

Let us estimate the performance of this idealized qubit-based protocol. Since the entanglement generation process (i)–(iii) is repeated until a single photon sent in step (ii) survives over the fibre transmission with transmittance 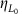, the average of the number *m* of channel uses to obtain the entanglement between adjacent nodes in step (iii) is 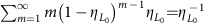. Hence, the idealized qubit-based protocol presents Alice and Bob a pair of qubits in a maximally entangled state by using 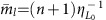 times of optical channels in total on average. Therefore, the average secret bits or ebits of the idealized qubit-based protocol per average total channel use is (*n*+1)^−1^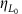, which is exactly the same scaling of the bound (3). This fact is also easily confirmed by seeing Fig. 2b.

Since the existing quantum repeater protocols^18^,^22^,^23^,^24^,^25^,^26^,^27^,^28^,^29^,^30^,^31^,^32^,^33^,^34^,^35^,^36^ are based on more practical devices than the idealized qubit-based protocol, they would be less efficient than the idealized qubit-based protocol, owing to more imperfections caused by the practical devices. However, there are schemes^27^,^28^,^29^,^32^,^34^,^35^ whose performance is essentially determined by distance *L*_0_ even under the use of such more practical devices similarly to the idealized qubit-based protocol as well as our bound (3). This implies that the quantum repeater protocols^27^,^28^,^29^,^32^,^34^,^35^ have no scaling gap with our bound (3).

### Upper bounds for DLCZ-type quantum repeaters

The bound (3) has been shown to be useful for understanding the ultimate performance of intercity QKD protocols and quantum repeater protocols. However, the original bound (2) for the general linear networks should have another fascinating applications beyond the purely lossy optical channel network. To show this, as an example, here we apply our bound to an exponential scaling problem^35^,^41^ of the DLCZ-type quantum repeater protocols^18^,^23^,^24^,^30^ with time-dependent decay of matter quantum memories. This problem was first pointed out by Razavi *et al*.^41^ by considering the practice of the matter quantum memories (although the DLCZ scheme was initially introduced^23^ as a protocol with polynomial scaling by assuming infinite coherence time of atomic-ensemble quantum memories). More precisely, Razavi *et al*. show that for the matter quantum memory with finite coherence time and no fault-tolerant protection the performance of the DLCZ-type protocols degrades exponentially with 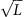, regardless of the used distillation scheme. However, we can obtain a more general and stronger result by using our bound (2). That is, from the bound (2), we can derive ultimate upper bounds on more general DLCZ-type quantum repeater protocols where even any single-shot quantum error correction for the matter quantum memories is allowed to be used in contrast to the paradigm of Razavi *et al*. Nonetheless, our bounds show that the coherence time of the matter quantum memories should be, at least, larger than 100 μs—which are comparable even with the up-to-date experimental result^42^ with retaining the coupling efficiency with photons—for enjoying the blessing of the DLCZ-type quantum repeaters.

Although the details can be found in Supplementary Note 2, here we present the main observation used to derive the upper bound for the DLCZ-type schemes. Conventionally, these schemes use the set of repeater nodes {*C*^*j*^}_*j*=1,2,…,2*n*+1_—which is composed of source repeater nodes {*C*^2*j*^}_*j*=1,2,…,*n*_ and receiver repeater nodes {*C*^2*j*+1^}_*j*=0,1,…,*n*_—between Alice *A*(=:*C*^0^) and Bob *B*(=:*C*^2*n*+2^), where *n*=2^*s*^−1 for *s*∈{0, 1, 2, …}. The source repeater nodes and the receiver repeater nodes are located alternately and at regular intervals, and the adjacent source nodes (adjacent receiver nodes) are separated over distance *L*_0_=*L*/(*n*+1). The unique feature of the DLCZ-type schemes is to use only probabilistic Bell measurements not only for the entanglement generation but also for the entanglement swapping, because the schemes adopt their implementation with linear optical elements and photon detectors by respecting the simplicity and practicality^18^,^23^,^24^,^30^. In particular, the schemes (Supplementary Fig. 1) begin with independent and parallel entanglement generation processes between adjacent source repeater nodes *C*^2*j*^ and *C*^2*j*+2^. These are accomplished by performing the Bell measurements at receiver node *C*^2*j*+1^ on pairs of optical pulses—each of which has been entangled with a matter quantum memory—from the adjacent nodes *C*^2*j*^ and *C*^2*j*+2^ over lossy optical channels 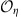 a transmittance *η*. Then, entangled pairs connecting source repeater nodes separated by 2^*i*^*L*_0_ are converted to ones separated by 2^*i*+1^*L*_0_ recursively (*i*=0, 1,…,*s*−1), until Alice and Bob share entangled pairs. This is done by sequential applications of the entanglement swapping to matter quantum memories in a knockout tournament manner over source repeater nodes {*C*^2*j*^}_*j*=1,2,…,*n*_. Here to perform the entanglement swapping as a step, source repeater node *C*^2*j*^ necessitates to receive heralding signals from distant repeater nodes to know which pairs of its own matter quantum memories should be subjected to the Bell measurements for the swapping. Hence, during the time *t*_2*j*_ from the beginning of entanglement generation to the arrival of the heralding signals, this repeater node *C*^2*j*^ needs to store entanglement in matter quantum memories with time-dependent decay modelled by a noisy qubit channel 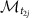. If we also respect the independence of the entanglement generation processes, as well as availability of only single-shot quantum error correction for matter quantum memories, the repeater node *C*^2*j*^ can thus be considered to be composed of three nodes 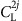, 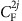 and 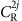. Here 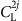 and 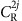 are connected to *C*^2*j*−1^ and *C*^2*j*+1^ by the lossy optical channel 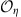 for the entanglement generation processes, respectively, and they are also linked by the noisy qubit channels 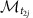 to 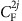 to perform the Bell measurements. Therefore, we can regard the DLCZ-type schemes as protocols working over a linear network (Supplementary Fig. 2) in the spacetime that is composed of vertices *V*={*A*, *C*^1^, 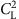, 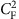, 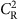, *C*^3^, …, *B*} connected by the lossy optical channels 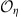 and the noisy qubit channels 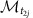. Since the minimum required memory time *t*_2*j*_ is determined by the location of the repeater node *C*^2*j*^ and the signalling time of the heralding signals, we can derive an upper bound on this linear network from equation (2) by deeming it as living merely in the space, rather than in the spacetime (Supplementary Note 2). Note that this implies that the upper bound may overestimate the performance of the DLCZ-type schemes, because the linear network over the space does not have any restriction^43^ coming from the arrow of time in contrast to that in the spacetime.

**Figure 3:**
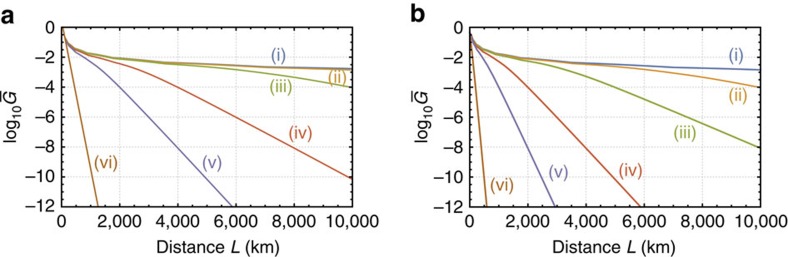
Upper bounds for DLCZ-type quantum repeaters with time-dependent memory decay. The performance of the protocols^18^,^23^,^24^,^30^ is measured in terms of the secret bits or ebits per average total channel use, 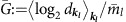, for the distance *L* between Alice and Bob. The protocols use repeater nodes {*C*^*j*^}_*j*=1,2,…,2*n*+1_ located at regular intervals and connected by optical fibres with transmittance 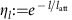 (*l*_att_=22 km) with each other, as in Fig. 1b. We assume that the coupling efficiency to the fibres is *η*_c_=0.9 and the speed of light in the fibres is *v*=2.0 × 10^8^ m s^−1^. The source nodes {*C*^2*j*^}_*j*=1,2,…,*n*_ are assumed to be equipped with matter quantum memories with dephasing, whose coherence time is (i) 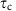
=1.0 × 10^−2^ s, (ii) 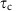
=5.0 × 10^−3^ s, (iii) 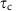
=2.5 × 10^−3^ s, (iv) 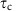
=1.0 × 10^−3^ s, (v) 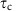
=5.0 × 10^−4^ s and (vi) 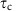
=1.0 × 10^−4^ s. The upper bounds for the protocols are obtained via being maximized over possible *n*. In **a** (In **b**), we show the upper bounds on the performance of the protocols for the application to QKD (for the application to entanglement distribution), where Alice and Bob do not need (Alice and Bob necessitate) to use matter quantum memories^35^. In **a** (In **b**), the upper bound (vi) is the same scaling of the intercity QKD protocols^19^,^20^,^21^ with the performance in the order of *η*_*L*/2_ (a point-to-point entanglement distribution protocol with the performance in the order of *η*_*L*_), implying that 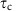
≤100 μs spoils the benefit to use the DLCZ-type quantum repeaters. Although these figures indicate that the upper bounds drastically improve with the coherence time 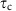 (>100 μs), this does not necessarily mean that there is a DLCZ-type scheme with similar performance, owing to the overestimation of the upper bounds.

In Fig. 3, we show the upper bounds on the linear network associated with the DLCZ-type quantum repeaters for the applications to the secret-key and entanglement generation between Alice and Bob. The difference between Fig. 3a and Fig. 3b stems from the fact that Alice and Bob need matter quantum memories for the case of the entanglement generation, while they do not for the case of the secret-key generation (see ref. 35 for instance). For the calculation of Fig. 3, the noisy qubit channel 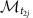 for the matter quantum memory is assumed to be modelled by a phase-flip channel with coherence time 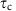. In addition, we suppose that the transmittance *η* of the lossy optical channel 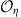 is described by 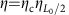 with the coupling efficiency *η*_c_ and the velocity of the heralding signals is equivalent to the speed *v* of light in optical fibres. Under these conditions, in Fig. 3, the number *n* associated with the number of repeater nodes is optimized to maximize the upper bounds. The existence of optimal *n* here—which is in contrast to the case for upper bounds for purely optical channel networks as in Fig. 2—stems from the existence of local errors/loss in the repeater nodes.

Despite these optimistic assumptions, Fig. 3 shows that even the upper bounds on the DLCZ-type quantum repeater schemes decay exponentially with the communication distance *L* for 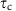
≤100 μs, although the threshold is comparable to the achieved coherence time in the up-to-date experiment^42^. This result may be reasonable by considering^35^ that the transmission time of the heralding signal over, for example, 100 km is already in the order of 100 μs. Although Fig. 3 indicates that the upper bounds drastically improve with the coherence time 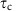 (≥100 μs), this does not necessarily mean that there is a DLCZ-type quantum repeater scheme with similar performance, owing to the overestimation of the upper bounds.

Of course, if we are allowed to repeat quantum error correction on the matter quantum memories while waiting for the heralding signals to arrive, then the coherence time of the matter quantum memories is not an issue. However, such a scheme to use such repeated quantum error correction cannot be called anymore the DLCZ-type quantum repeater protocols^18^,^23^,^24^,^30^ respecting the practical simplicity.

## Discussion

We have presented a fundamental upper bound (1) on the performance of any two-party quantum communication scheme over arbitrary quantum network topology. Besides, we have focused on its application to the general linear quantum network. As a result, we have seen that the bound (2) for the linear network is powerful enough to present rate-loss trade-offs (3) with the same scaling as existing intercity QKD protocols and quantum repeaters. However, the goodness of our bound (1) should not be restricted only to linear networks. In fact, very recently, Azuma and Kato^44^ have proposed a scheme that runs quantum repeater protocols between Alice and Bob in parallel over any given network, and they have shown that it has no scaling gap with our upper bound (1) for the case of lossy optical channel networks, irrespectively of the network topology. Since each of the quantum repeater protocols in this scheme is merely performed over a linear network, this protocol implies that it is important to optimize quantum repeater protocols via comparing its performance with our bound (2) for the linear network. More importantly, that fact suggests that our bound (1) is strong enough to evaluate the goodness of any protocol working over a general optical quantum network beyond linear ones.

In addition, we have treated a quantum internet protocol as if it supplies only a pair of clients, called Alice *A* and Bob *B*, with secret bits or ebits. Here we highlight that in fact our bound applies to multiple-pair cases where multiple pairs of parties try to establish secret bits or ebits at the same time. Suppose that there are *m* pairs of clients labelled by an index *j* so that a node *A*^*j*^∈*V* would like to share secret bits or ebits with another node *B*^*j*^∈*V* for *j*=1, 2, …, *m* by using a quantum network associated with a graph *G*=(*V*, *E*). Then, if a quantum internet protocol presents pair *A*^*j*^*B*^*j*^ with 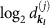 secret bits or ebits within an error 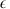
(>0) with probability 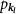 for all *j*=1, 2, …, *m*, the protocol obeys the following bound, which can be obtained similarly to equation (1) (see the proof in Supplementary Note 3): for any *V*′⊂*V*, we have


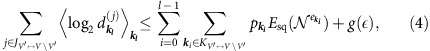


where we write 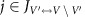 when *A*^*j*^∈*V*′ and *B*^*j*^∈*V*\*V*′ or when *B*^*j*^∈*V*′ and *A*^*j*^∈*V*\*V*′. Therefore, our bound is applied to any multi-pair bipartite quantum communication protocol.

Despite the generalized bound (4), we have still focused on bipartite quantum communication protocols over a given network. However, our bound (1) is applicable even to any multi-party protocol^16^,^45^ based on sharing a multipartite resource^46^—such as a multipartite private key^47^ or a multipartite entangled state like a Greenberger–Horne–Zeilinger state and a cluster state—among plural clients. This is because such a multipartite resource is, usually, freely transformed into a corresponding bipartite resource—secret bits or ebits—between any two of the clients by using an additional LOCC operation, to which our bound (1) is applied. Therefore, our bound should provide an upper bound even to such a multi-party quantum communication protocol.

We have also shown how to associate a class of practical quantum repeater protocols, called DLCZ-type quantum repeaters, with a linear quantum network composed of noisy qubit channels and lossy optical channels—corresponding to the models for matter quantum memories and optical fibres, respectively. Besides, by regarding the noisy qubit channels as the phase-flip channels for simplicity, from the upper bound (2) on the linear network, we have concluded that the coherence time of matter quantum memories should be, at least, longer than 100 μs to enjoy the blessing of the DLCZ-type quantum repeater schemes. However, this kind of correspondence between a practical quantum information processing (QIP) protocol and a quantum network is not unique, and it should have degrees of freedom a lot enough to derive good upper bounds on the performance of various kinds of QIP protocols. In particular, by finding out a proper correspondence between a given QIP protocol and a quantum network, our bounds (1) and (4) should present a fundamental upper bound, from which we can derive a non-trivial conclusion like the minimum coherence time required by the DLCZ-type quantum repeater schemes. For example, our bounds (1) and (4) would be useful for deriving the ultimate performance of the distributed quantum computation^30^,^48^,^49^,^50^,^51^ and of more practical quantum repeaters with more complicated noise models. This versatility of our bounds would be in contrast to Pirandola's bound^37^ restricted to teleportation stretchable quantum channel networks. This is because one would not be surprised if a practical QIP scheme involves quantum channels without teleportation stretchability.

While we have used mainly the TGW bound in our paper, it should be noted that our reduction idea is useful^44^ for deriving a good bound for a general network topology from a bound for point-to-point quantum communication generally. We have just begun to grasp full implications of our bound (1): for instance, its tighter version for specific channels like Pirandola's one^37^ or with deriving a better bound^52^ for the squashed entanglement of the channel, its applications to the many-body quantum physics in any spacetime topology regarded as a quantum network^1^ and to a more complicated quantum communication channel network—such as a multi-party protocol with broadcasting channels^53^,^54^,^55^—will be in a fair way to appear.

## Methods

### Upper bound (2) for the general linear network

Here we derive the bound (2) from the general bound (1). Since secret bits or ebits obtained through any quantum internet protocol must obey the bound (1), any scheme working over the general linear network should follow


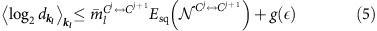


for the choice of *V*_*A*_={*C*^0^, …, *C*^*j*^} with *j*=0, 1, …, *n*, where 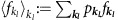 and 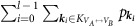 for the choice of *V*_*A*_={*C*^0^, …, *C*^*j*^} is rephrased as the average number 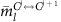 of times the quantum channel between nodes *C*^*j*^ and *C*^*j*+1^ is used. Since equation (5) holds for any *j*=0, 1, …, *n*, obtained secret bits or ebits are most tightly bounded as


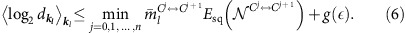


By assuming that we can freely choose 
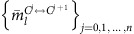
 to maximize 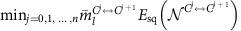 with the average total number 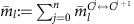 of channel uses fixed, we have


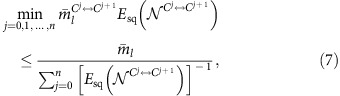


where the equality holds when 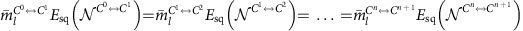. This formulation highlights a difference in the performance measure from Pirandola's one^37^ based on the restriction of 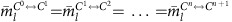. Combining equation (7) with equation (6), we obtain the bound (2).

### Data availability

The data that support the findings of this study are available from the corresponding author on request.

## Additional information

**How to cite this article:** Azuma, K. *et al*. Fundamental rate-loss trade-off for the quantum internet. *Nat. Commun.* **7,** 13523 doi: 10.1038/ncomms13523 (2016).

**Publisher's note**: Springer Nature remains neutral with regard to jurisdictional claims in published maps and institutional affiliations.

## Supplementary Material

Supplementary Information Supplementary Figures 1-2, Supplementary Notes 1-3 and Supplementary References.

Peer Review File
