# Supplementary material for: Fundamental rate-loss trade-off for the quantum internet
Source: Nat Commun. 2016 Nov 25;7:13523. doi: 10.1038/ncomms13523 (PMC5133617; doi:10.1038/ncomms13523)
Supplement: Supplementary Information — Supplementary Figures 1-2, Supplementary Notes 1-3 and Supplementary References. [file ncomms13523-s1.pdf]

Supplementary Figures

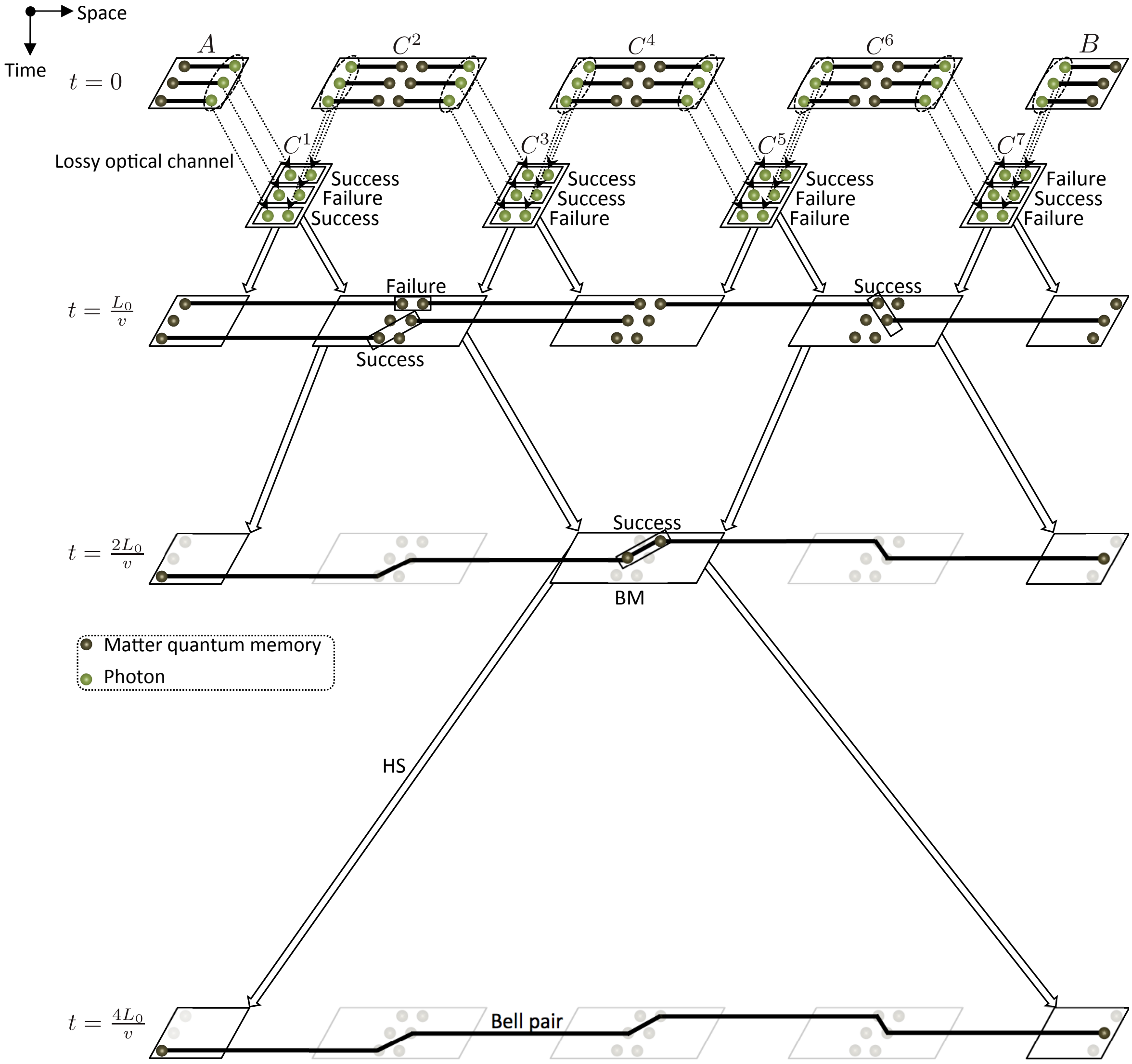

Supplementary Figure 1: DLCZ-type quantum repeater schemes with repeater nodes $\{C^j\}_{j=1,2,\ldots,2n+1}$ ($n = 3$ here). The feature of those schemes is to use only *probabilistic* Bell measurements (BMs) in *any* stage. In particular, at time $t = 0$, the schemes begin with independent and parallel entanglement generation processes between adjacent repeater nodes C^{2j} and C^{2j+2}. These are accomplished by performing the Bell measurements at a repeater node C^{2j+1} on pairs of optical pulses—each of which has been entangled with a matter quantum memory—from adjacent nodes C^{2j} and C^{2j+2} over lossy optical channels $\mathcal{O}_\eta$. This is followed by sequential applications of the entanglement swapping in a knockout tournament manner to matter quantum memories to obtain entangled pairs over a longer distance by connecting two (short) entangled pairs. In any stage, having performed the BMs, a repeater node needs to send the measurement outcomes about successes/failures as the heralding signals (HSs) to another distant repeater nodes in order to instruct them on which pairs of matter quantum memories should be subjected to the BMs as the next step. The velocity of the HSs should be upper bounded by the speed v of light in optical fibres.

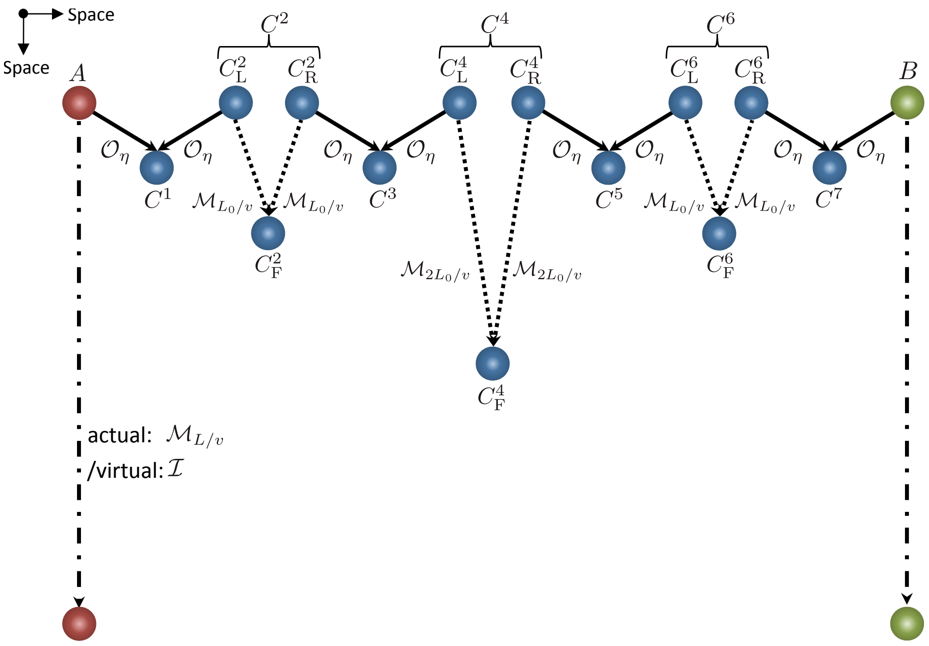

Supplementary Figure 2: Linear network associated with the DLCZ-type quantum repeater schemes in Supplementary Fig. 1. This network is composed of vertices $V = \{A, C^1, C^2_{\rm L}, C^2_{\rm F}, C^2_{\rm R}, C^3, \ldots, C^{2n-1}, C^{2n}_{\rm L}, C^{2n}_{\rm F}, C^{2n}_{\rm R}, C^{2n+1}, B\}$ connected by the noisy channels $\{\mathcal{M}_{t_{2j}}\}_{j=1,2,\ldots,n}$ and lossy optical channels $\mathcal{O}_\eta$ ($n = 3$ here). Note that Alice and Bob need matter quantum memories with time-dependent decay for the case of the entanglement generation, while they do not for the case of the secret-key generation.

Supplementary Note 1. PROOF FOR THE MAIN RESULT (1)

Here we provide the proof for the main result, that is, Eq. (1). Although here we focus on deriving the bound (1) for QKD protocols between Alice and Bob, the same technique can be applied for protocols to share entanglement between them, similarly to the TGW bound [1, 2].

Suppose that, with help of other parties $\{C^j\}_{j=1,2,\dots,n}$ in a quantum network, Alice and Bob share physical systems $\mathcal{H}_{A'A''} \otimes \mathcal{H}_{B'B''}$ in private state [3]

$$\hat{\gamma}_d^{AB} = \hat{U}^{A'B'A''B''}(|\Phi_d\rangle\langle\Phi_d|_{A'B'} \otimes \hat{\rho}^{A''B''})\hat{U}^{A'B'A''B''\dagger} \tag{1.1}$$

with unitary operator $\hat{U}^{A'B'A''B''} := \sum_{i,j=0}^{d-1} |ij\rangle\langle ij|_{A'B'} \otimes \hat{U}_{ij}^{A''B''}$, maximally entangled state $|\Phi_d\rangle_{A'B'} := \sum_{i=0}^{d-1} |ii\rangle_{A'B'}/\sqrt{d}$ and orthonormal states $\{|ij\rangle_{A'B'}\}_{i,j=0,1,\dots,d-1}$ for systems $\mathcal{H}_{A'} \otimes \mathcal{H}_{B'}$. In particular, Alice and Bob obtain a private state through the following most general adaptive protocol: (i) Alice, Bob and parties $\{C^j\}_{j=1,2,\dots,n}$ begin by preparing their physical system $\mathcal{H}^0$ in a separable state $\hat{\rho}_1^{ABC^1C^2\dots C^n}$, where

$$\mathcal{H}^j := \mathcal{H}_A^j \otimes \mathcal{H}_B^j \otimes \mathcal{H}_{C^1}^j \otimes \mathcal{H}_{C^2}^j \otimes \cdots \otimes \mathcal{H}_{C^n}^j \tag{1.2}$$

and $\mathcal{H}_X^j$ represents the physical system held by party $X \in \{A, B, C^1, C^2, \dots, C^n\}$. (ii) In the first round, party $X|1 \in \{A, B, C^1, C^2, \dots, C^n\}$ sends his/her subsystem $\bar{\mathcal{H}}_{X|1}$ to another party $Y|1$ through quantum channel $\mathcal{N}^{\bar{\mathcal{H}}_{X|1} \to \tilde{\mathcal{H}}_{Y|1}}$ with isometric extension $\mathcal{U}^{\bar{\mathcal{H}}_{X|1} \to \tilde{\mathcal{H}}_{Y|1} \otimes \mathcal{H}_{E_1}}$ for environment system $\mathcal{H}_{E_1}$, which provides a refreshed description of the whole system, $\mathcal{H}^{0'|1}$ with subsystems $\mathcal{H}_{Y|1}^{0'|1} = \mathcal{H}_{Y|1}^0 \otimes \tilde{\mathcal{H}}_{Y|1}$, $\mathcal{H}_{X|1}^0 = \mathcal{H}_{X|1}^{0'|1} \otimes \bar{\mathcal{H}}_{X|1}$ and $\mathcal{H}_Z^{0'|1} = \mathcal{H}_Z^0$ for any party Z except for parties $X|1$ and $Y|1$. This is followed by an LOCC operation, which presents a renewed entire system $\mathcal{H}^{1|1}$ in state $\hat{\rho}_{k_1}^{ABC^1C^2\dots C^n}$ with probability p_{k_1}. Let $\mathcal{H}_{R_{k_1}}$ be a system that purifies the state $\hat{\rho}_{k_1}^{ABC^1C^2\dots C^n}$, providing pure-state expression $|\rho_{k_1}\rangle_{ABC^1C^2\dots C^n R_{k_1}}$. (iii) Similarly, in the ith round ($i = 2, 3, \dots, l$), depending on the previous outcomes $\boldsymbol{k}_{i-1} := k_{i-1} \cdots k_1$ (with $\boldsymbol{k}_0 := 1$), for given entire system $\mathcal{H}^{(i-1)|\boldsymbol{k}_{i-2}}$, party $X|\boldsymbol{k}_{i-1} \in \{A, B, C^1, C^2, \dots, C^n\}$ may send his/her subsystem $\bar{\mathcal{H}}_{X|\boldsymbol{k}_{i-1}}$ to another party $Y|\boldsymbol{k}_{i-1} \in \{A, B, C^1, C^2, \dots, C^n\}$ through quantum channel $\mathcal{N}^{\bar{\mathcal{H}}_{X|\boldsymbol{k}_{i-1}} \to \tilde{\mathcal{H}}_{Y|\boldsymbol{k}_{i-1}}}$ with isometric extension $\mathcal{U}^{\bar{\mathcal{H}}_{X|\boldsymbol{k}_{i-1}} \to \tilde{\mathcal{H}}_{Y|\boldsymbol{k}_{i-1}} \otimes \mathcal{H}_{E_{\boldsymbol{k}_{i-1}}}}$ for environment system $\mathcal{H}_{E_{\boldsymbol{k}_{i-1}}}$, which updates the description of the whole system as $\mathcal{H}^{(i-1)'|\boldsymbol{k}_{i-1}}$ with $\mathcal{H}_{Y|\boldsymbol{k}_{i-1}}^{(i-1)'|\boldsymbol{k}_{i-1}} = \mathcal{H}_{Y|\boldsymbol{k}_{i-1}}^{(i-1)|\boldsymbol{k}_{i-2}} \otimes \tilde{\mathcal{H}}_{Y|\boldsymbol{k}_{i-1}}$, $\mathcal{H}_{X|\boldsymbol{k}_{i-1}}^{(i-1)|\boldsymbol{k}_{i-2}} = \mathcal{H}_{X|\boldsymbol{k}_{i-1}}^{(i-1)'|\boldsymbol{k}_{i-1}} \otimes \bar{\mathcal{H}}_{X|\boldsymbol{k}_{i-1}}$ and $\mathcal{H}_Z^{(i-1)'|\boldsymbol{k}_{i-1}} = \mathcal{H}_Z^{(i-1)|\boldsymbol{k}_{i-2}}$ for any party Z except for parties $X|\boldsymbol{k}_{i-1}$ and $Y|\boldsymbol{k}_{i-1}$. This is followed by an LOCC operation, providing an entire system $\mathcal{H}^{i|\boldsymbol{k}_{i-1}}$ in state $\hat{\rho}_{\boldsymbol{k}_i}^{ABC^1C^2\dots C^n}$ with probability $p_{k_i|\boldsymbol{k}_{i-1}}$. Let $\mathcal{H}_{R_{\boldsymbol{k}_i}}$ be a system that purifies the state $\hat{\rho}_{\boldsymbol{k}_i}^{ABC^1C^2\dots C^n}$, presenting pure-state expression $|\rho_{\boldsymbol{k}_i}\rangle_{ABC^1C^2\dots C^n R_{\boldsymbol{k}_i}}$. (iv) Finally, i.e., in the lth round, Alice and Bob obtain state $\hat{\rho}_{\boldsymbol{k}_l}^{ABC^1C^2\dots C^n}$ close to private state $\hat{\gamma}_{d_{\boldsymbol{k}_l}}^{AB}$ for integer $d_{\boldsymbol{k}_l} (\geq 1)$.

From the definition, the final state $\hat{\rho}_{\boldsymbol{k}_l}^{ABC^1C^2\dots C^n}$ should be close to private state $\hat{\gamma}_{d_{\boldsymbol{k}_l}}^{AB}$, i.e., $\|\hat{\rho}_{\boldsymbol{k}_l}^{AB} - \hat{\gamma}_{d_{\boldsymbol{k}_l}}^{AB}\|_1 \leq \epsilon$ for $\epsilon > 0$, where we define $\hat{\rho}^X := \mathrm{Tr}_Y(\hat{\rho}^{XY})$. Then, from Theorem 2 in Ref. [4], we have

$$\log_2 d_{\boldsymbol{k}_l} \leq E_{\mathrm{sq}}^{\mathcal{H}_A^{l|\boldsymbol{k}_{l-1}} : \mathcal{H}_B^{l|\boldsymbol{k}_{l-1}}}(\hat{\rho}_{\boldsymbol{k}_l}^{AB}) + g(\epsilon) \tag{1.3}$$

with a continuous function $g(\epsilon)$ with the property of $\lim_{\epsilon \to 0} g(\epsilon) = 0$ and the squashed entanglement $E_{\mathrm{sq}}^{X:Y}(\hat{\rho}^{XY})$ between systems X and Y in state $\hat{\rho}^{XY}$ [5]. Note that the function $g(\epsilon)$ for the private-key distillation could be different from that for the case of entanglement distillation [1, 2], but, in both cases, $\lim_{\epsilon \to 0} g(\epsilon) = 0$ holds [1, 2, 4].

Our proof for Eq. (1) is made by regarding the general multi-party protocol as bipartite communication and by applying the technique of the TGW bound [1] to the bipartite one. Hence, let us divide the set of parties $\{A, B, C^1, C^2, \dots, C^n\} (=: V)$ into two disjoint groups V_A and $V_B (= V \setminus V_A)$ that include parties A and B, respectively. We define $\mathcal{H}_{V_A}^r := \otimes_{X \in V_A} \mathcal{H}_X^r$ and $\mathcal{H}_{V_B}^r := \otimes_{X \in V_B} \mathcal{H}_X^r$. In addition, for given $\boldsymbol{k}_{i-1}$, if we use quantum channel $\mathcal{N}^{\bar{\mathcal{H}}_{X|\boldsymbol{k}_{i-1}} \to \tilde{\mathcal{H}}_{Y|\boldsymbol{k}_{i-1}}}$ with $X|\boldsymbol{k}_{i-1} \in V_C$ and $Y|\boldsymbol{k}_{i-1} \in V \setminus V_C$ for $C = A$ or $C = B$, we write $\boldsymbol{k}_{i-1} \in K_{V_A \leftrightarrow V_B}$. In what follows, we derive inequalities for two cases, $\boldsymbol{k}_{i-1} \notin K_{V_A \leftrightarrow V_B}$ and $\boldsymbol{k}_{i-1} \in K_{V_A \leftrightarrow V_B}$.

Let us consider the case of $\boldsymbol{k}_{i-1} \notin K_{V_A \leftrightarrow V_B}$, which is a trivial case. This case corresponds to either a situation where we do not use quantum channels or a situation where we use quantum channel $\mathcal{N}^{\bar{\mathcal{H}}_{X|\boldsymbol{k}_{i-1}} \to \tilde{\mathcal{H}}_{Y|\boldsymbol{k}_{i-1}}}$ with $X|\boldsymbol{k}_{i-1} \in V_C$ and $Y|\boldsymbol{k}_{i-1} \in V_C$ for $C = A$ or $C = B$. In the former case, since the squashed entanglement does not increase on

average under partial traces and LOCC, we have

$$\begin{aligned}\sum_{k_i} p_{k_i|\boldsymbol{k}_{i-1}} E_{\mathrm{sq}}^{\mathcal{H}_A^{i|\boldsymbol{k}_{i-1}}:\mathcal{H}_B^{i|\boldsymbol{k}_{i-1}}}(\hat{\rho}_{\boldsymbol{k}_i}^{AB}) \le& \sum_{k_i} p_{k_i|\boldsymbol{k}_{i-1}} E_{\mathrm{sq}}^{\mathcal{H}_{V_A}^{i|\boldsymbol{k}_{i-1}}:\mathcal{H}_{V_B}^{i|\boldsymbol{k}_{i-1}}}(\hat{\rho}_{\boldsymbol{k}_i}^{ABC^1C^2\ldots C^n}) && (1.4)\\ \le& E_{\mathrm{sq}}^{\mathcal{H}_{V_A}^{(i-1)|\boldsymbol{k}_{i-2}}:\mathcal{H}_{V_B}^{(i-1)|\boldsymbol{k}_{i-2}}}(\hat{\rho}_{\boldsymbol{k}_{i-1}}^{ABC^1C^2\ldots C^n}). && (1.5)\end{aligned}$$

Even in the latter case, the channel $\mathcal{N}^{\bar{\mathcal{H}}_{X|\boldsymbol{k}_{i-1}}\to\tilde{\mathcal{H}}_{Y|\boldsymbol{k}_{i-1}}}$ should be regarded as just a local channel for the bipartite communication between V_A and V_B. To make this clearer, let us first assume $X|\boldsymbol{k}_{i-1}\in V_A$ and $Y|\boldsymbol{k}_{i-1}\in V_A$. Then, we have

$$\begin{aligned}\sum_{k_i} p_{k_i|\boldsymbol{k}_{i-1}} E_{\mathrm{sq}}^{\mathcal{H}_A^{i|\boldsymbol{k}_{i-1}}:\mathcal{H}_B^{i|\boldsymbol{k}_{i-1}}}(\hat{\rho}_{\boldsymbol{k}_i}^{AB}) \le& \sum_{k_i} p_{k_i|\boldsymbol{k}_{i-1}} E_{\mathrm{sq}}^{\mathcal{H}_{V_A}^{i|\boldsymbol{k}_{i-1}}:\mathcal{H}_{V_B}^{i|\boldsymbol{k}_{i-1}}}(\hat{\rho}_{\boldsymbol{k}_i}^{ABC^1C^2\ldots C^n}) && (1.6)\\ \le& E_{\mathrm{sq}}^{\mathcal{H}_{V_A}^{(i-1)'|\boldsymbol{k}_{i-1}}:\mathcal{H}_{V_B}^{(i-1)'|\boldsymbol{k}_{i-1}}}(\mathcal{N}^{\bar{\mathcal{H}}_{X|\boldsymbol{k}_{i-1}}\to\tilde{\mathcal{H}}_{Y|\boldsymbol{k}_{i-1}}}(\hat{\rho}_{\boldsymbol{k}_{i-1}}^{ABC^1C^2\ldots C^n})) && (1.7)\\ \le& E_{\mathrm{sq}}^{\mathcal{H}_{V_A}^{(i-1)|\boldsymbol{k}_{i-2}}:\mathcal{H}_{V_B}^{(i-1)|\boldsymbol{k}_{i-2}}}(\hat{\rho}_{\boldsymbol{k}_{i-1}}^{ABC^1C^2\ldots C^n}). && (1.8)\end{aligned}$$

The first inequality is derived from the fact that the squashed entanglement does not increase under partial traces. The second inequality comes from the fact that the squashed entanglement does not increase on average under LOCC. The final inequality states that the squashed entanglement does not increase under any local quantum channel. The same inequality is obtained if we begin by assuming $X|\boldsymbol{k}_{i-1}\in V_B$ and $Y|\boldsymbol{k}_{i-1}\in V_B$. Therefore, $\boldsymbol{k}_{i-1}\notin K_{V_A\leftrightarrow V_B}$ finally presents Eq. (1.5) in any case.

Let us consider the case of $\boldsymbol{k}_{i-1}\in K_{V_A\leftrightarrow V_B}$. In this case, $\mathcal{N}^{\bar{\mathcal{H}}_{X|\boldsymbol{k}_{i-1}}\to\tilde{\mathcal{H}}_{Y|\boldsymbol{k}_{i-1}}}$ is a channel connecting parties V_A and V_B nontrivially, which should put a limitation on the communication. To make this more precise, we first assume $X|\boldsymbol{k}_{i-1}\in V_A$ and $Y|\boldsymbol{k}_{i-1}\in V_B$. Then, we have

$$\begin{aligned}&\sum_{k_i} p_{k_i|\boldsymbol{k}_{i-1}} E_{\mathrm{sq}}^{\mathcal{H}_A^{i|\boldsymbol{k}_{i-1}}:\mathcal{H}_B^{i|\boldsymbol{k}_{i-1}}}(\hat{\rho}_{\boldsymbol{k}_i}^{AB}) \le \sum_{k_i} p_{k_i|\boldsymbol{k}_{i-1}} E_{\mathrm{sq}}^{\mathcal{H}_{V_A}^{i|\boldsymbol{k}_{i-1}}:\mathcal{H}_{V_B}^{i|\boldsymbol{k}_{i-1}}}(\hat{\rho}_{\boldsymbol{k}_i}^{ABC^1C^2\ldots C^n}) && (1.9)\\ &\le E_{\mathrm{sq}}^{\mathcal{H}_{V_A}^{(i-1)'|\boldsymbol{k}_{i-1}}:\mathcal{H}_{V_B}^{(i-1)'|\boldsymbol{k}_{i-1}}}(\mathcal{N}^{\bar{\mathcal{H}}_{X|\boldsymbol{k}_{i-1}}\to\tilde{\mathcal{H}}_{Y|\boldsymbol{k}_{i-1}}}(\hat{\rho}_{\boldsymbol{k}_{i-1}}^{ABC^1C^2\ldots C^n})) && (1.10)\\ &= E_{\mathrm{sq}}^{\mathcal{H}_{V_A}^{(i-1)'|\boldsymbol{k}_{i-1}}:\mathcal{H}_{V_B\setminus(Y|\boldsymbol{k}_{i-1})}^{(i-1)'|\boldsymbol{k}_{i-1}}\otimes\mathcal{H}_{Y|\boldsymbol{k}_{i-1}}^{(i-1)|\boldsymbol{k}_{i-2}}\otimes\tilde{\mathcal{H}}_{Y|\boldsymbol{k}_{i-1}}}(\mathcal{U}^{\bar{\mathcal{H}}_{X|\boldsymbol{k}_{i-1}}\to\tilde{\mathcal{H}}_{Y|\boldsymbol{k}_{i-1}}\otimes\mathcal{H}_{E_{\boldsymbol{k}_{i-1}}}}(|\rho_{\boldsymbol{k}_{i-1}}\rangle_{ABC^1C^2\ldots C^nR_{\boldsymbol{k}_{i-1}}})) && (1.11)\\ &\le E_{\mathrm{sq}}^{\mathcal{H}_{V_A}^{(i-1)'|\boldsymbol{k}_{i-1}}\otimes\mathcal{H}_{V_B\setminus(Y|\boldsymbol{k}_{i-1})}^{(i-1)'|\boldsymbol{k}_{i-1}}\otimes\mathcal{H}_{Y|\boldsymbol{k}_{i-1}}^{(i-1)|\boldsymbol{k}_{i-2}}\otimes\mathcal{H}_{R_{\boldsymbol{k}_{i-1}}}:\tilde{\mathcal{H}}_{Y|\boldsymbol{k}_{i-1}}}(\mathcal{U}^{\bar{\mathcal{H}}_{X|\boldsymbol{k}_{i-1}}\to\tilde{\mathcal{H}}_{Y|\boldsymbol{k}_{i-1}}\otimes\mathcal{H}_{E_{\boldsymbol{k}_{i-1}}}}(|\rho_{\boldsymbol{k}_{i-1}}\rangle_{ABC^1C^2\ldots C^nR_{\boldsymbol{k}_{i-1}}}))\\ &\quad + E_{\mathrm{sq}}^{\mathcal{H}_{V_A}^{(i-1)'|\boldsymbol{k}_{i-1}}\otimes\tilde{\mathcal{H}}_{Y|\boldsymbol{k}_{i-1}}\otimes\mathcal{H}_{E_{\boldsymbol{k}_{i-1}}}:\mathcal{H}_{V_B\setminus(Y|\boldsymbol{k}_{i-1})}^{(i-1)'|\boldsymbol{k}_{i-1}}\otimes\mathcal{H}_{Y|\boldsymbol{k}_{i-1}}^{(i-1)|\boldsymbol{k}_{i-2}}}(\mathcal{U}^{\bar{\mathcal{H}}_{X|\boldsymbol{k}_{i-1}}\to\tilde{\mathcal{H}}_{Y|\boldsymbol{k}_{i-1}}\otimes\mathcal{H}_{E_{\boldsymbol{k}_{i-1}}}}(|\rho_{\boldsymbol{k}_{i-1}}\rangle_{ABC^1C^2\ldots C^nR_{\boldsymbol{k}_{i-1}}})) && (1.12)\\ &= E_{\mathrm{sq}}^{\mathcal{H}_{V_A}^{(i-1)'|\boldsymbol{k}_{i-1}}\otimes\mathcal{H}_{V_B\setminus(Y|\boldsymbol{k}_{i-1})}^{(i-1)|\boldsymbol{k}_{i-2}}\otimes\mathcal{H}_{Y|\boldsymbol{k}_{i-1}}^{(i-1)|\boldsymbol{k}_{i-2}}\otimes\mathcal{H}_{R_{\boldsymbol{k}_{i-1}}}:\tilde{\mathcal{H}}_{Y|\boldsymbol{k}_{i-1}}}(\mathcal{N}^{\bar{\mathcal{H}}_{X|\boldsymbol{k}_{i-1}}\to\tilde{\mathcal{H}}_{Y|\boldsymbol{k}_{i-1}}}(|\rho_{\boldsymbol{k}_{i-1}}\rangle_{ABC^1C^2\ldots C^nR_{\boldsymbol{k}_{i-1}}}))\\ &\quad + E_{\mathrm{sq}}^{\mathcal{H}_{V_A}^{(i-1)'|\boldsymbol{k}_{i-1}}\otimes\bar{\mathcal{H}}_{X|\boldsymbol{k}_{i-1}}:\mathcal{H}_{V_B\setminus(Y|\boldsymbol{k}_{i-1})}^{(i-1)|\boldsymbol{k}_{i-2}}\otimes\mathcal{H}_{Y|\boldsymbol{k}_{i-1}}^{(i-1)|\boldsymbol{k}_{i-2}}}(|\rho_{\boldsymbol{k}_{i-1}}\rangle_{ABC^1C^2\ldots C^nR_{\boldsymbol{k}_{i-1}}}) && (1.13)\\ &\le E_{\mathrm{sq}}(\mathcal{N}^{\bar{\mathcal{H}}_{X|\boldsymbol{k}_{i-1}}\to\tilde{\mathcal{H}}_{Y|\boldsymbol{k}_{i-1}}}) + E_{\mathrm{sq}}^{\mathcal{H}_{V_A}^{(i-1)|\boldsymbol{k}_{i-2}}:\mathcal{H}_{V_B}^{(i-1)|\boldsymbol{k}_{i-2}}}(\hat{\rho}_{\boldsymbol{k}_{i-1}}^{ABC^1C^2\ldots C^n}). && (1.14)\end{aligned}$$

The first inequality is derived from the fact that the squashed entanglement does not increase under partial traces. The second inequality comes from the fact that the squashed entanglement does not decrease on average under LOCC. The third inequality is the application of Lemma 2 in Ref. [1] by regarding $\mathcal{H}_{V_A}^{(i-1)'|\boldsymbol{k}_{i-1}}$ as system A, $\tilde{\mathcal{H}}_{Y|\boldsymbol{k}_{i-1}}$ as system B_1, $\mathcal{H}_{E_{\boldsymbol{k}_{i-1}}}$ as system E_1, $\mathcal{H}_{V_B\setminus(Y|\boldsymbol{k}_{i-1})}^{(i-1)'|\boldsymbol{k}_{i-1}}\otimes\mathcal{H}_{Y|\boldsymbol{k}_{i-1}}^{(i-1)|\boldsymbol{k}_{i-2}}$ as system B_2, and $\mathcal{H}_{R_{\boldsymbol{k}_{i-1}}}$ as system E_2. The final inequality follows from the definition [1] of the squashed entanglement of a quantum channel. The same inequality is derived if we start by assuming $X|\boldsymbol{k}_{i-1}\in V_B$ and $Y|\boldsymbol{k}_{i-1}\in V_A$.

Therefore, using Eqs. (1.5) and (1.14) recursively and the fact that $\hat{\rho}_1^{ABC^1C^2\ldots C^n}$ is separable, we obtain

$$\sum_{\boldsymbol{k}_l} p_{\boldsymbol{k}_l} E_{\rm sq}^{\mathcal{H}_A^{l|\boldsymbol{k}_{l-1}}:\mathcal{H}_B^{l|\boldsymbol{k}_{l-1}}}(\hat{\rho}_{\boldsymbol{k}_l}^{AB}) = \sum_{\boldsymbol{k}_{l-1}} p_{\boldsymbol{k}_{l-1}} \sum_{k_l} p_{k_l|\boldsymbol{k}_{l-1}} E_{\rm sq}^{\mathcal{H}_A^{l|\boldsymbol{k}_{l-1}}:\mathcal{H}_B^{l|\boldsymbol{k}_{l-1}}}(\hat{\rho}_{\boldsymbol{k}_l}^{AB}) \tag{1.15}$$

$$\leq \sum_{\boldsymbol{k}_{l-1}\in K_{V_A\leftrightarrow V_B}} p_{\boldsymbol{k}_{l-1}} E_{\rm sq}(\mathcal{N}^{\tilde{\mathcal{H}}_{X|\boldsymbol{k}_{l-1}}\to\tilde{\mathcal{H}}_{Y|\boldsymbol{k}_{l-1}}})$$

$$+\sum_{\boldsymbol{k}_{l-1}} p_{\boldsymbol{k}_{l-1}} E_{\rm sq}^{\mathcal{H}_{V_A}^{(l-1)|\boldsymbol{k}_{l-2}}:\mathcal{H}_{V_B}^{(l-1)|\boldsymbol{k}_{l-2}}}(\hat{\rho}_{\boldsymbol{k}_{l-1}}^{ABC^1C^2\ldots C^n}) \tag{1.16}$$

$$\leq \sum_{i=1}^{l} \sum_{\boldsymbol{k}_{i-1}\in K_{V_A\leftrightarrow V_B}} p_{\boldsymbol{k}_{i-1}} E_{\rm sq}(\mathcal{N}^{\tilde{\mathcal{H}}_{X|\boldsymbol{k}_{i-1}}\to\tilde{\mathcal{H}}_{Y|\boldsymbol{k}_{i-1}}}). \tag{1.17}$$

Combined with Eq. (1.3), this concludes

$$\sum_{\boldsymbol{k}_l} p_{\boldsymbol{k}_l} \log_2 d_{\boldsymbol{k}_l} \leq \sum_{i=1}^{l} \sum_{\boldsymbol{k}_{i-1}\in K_{V_A\leftrightarrow V_B}} p_{\boldsymbol{k}_{i-1}} E_{\rm sq}(\mathcal{N}^{\tilde{\mathcal{H}}_{X|\boldsymbol{k}_{i-1}}\to\tilde{\mathcal{H}}_{Y|\boldsymbol{k}_{i-1}}}) + g(\epsilon). \tag{1.18}$$

This is equivalent to Eq. (1).

Supplementary Note 2. UPPER BOUNDS ON DLCZ-TYPE QUANTUM REPEATER PROTOCOLS WITH TIME-DEPENDENT MEMORY DECAY

Here we show that Duan-Lukin-Cirac-Zoller-type (DLCZ-type) quantum repeater schemes [6–9] with time-dependent decay of matter quantum memories can be regarded as special cases of the general linear quantum network. Besides, we present theoretical upper bounds on those schemes, which provide how much coherence time of matter quantum memories is, *at least*, needed to make those schemes useful.

Let us start by reviewing a typical setup of quantum repeaters. As can be seen in Supplementary Fig. 1, conventionally, in quantum repeater protocols, all the repeater nodes $\{C^j\}_{j=1,2,\ldots,2n+1}$ between Alice and Bob are classified into two sets, source repeater nodes $\{C^{2j}\}_{j=1,\ldots,n}$ and receiver repeater nodes $\{C^{2j+1}\}_{j=0,1,\ldots,n}$, and the source repeater nodes and the receiver repeater nodes are located alternately and at regular intervals. This implies that, for a given distance L between Alice and Bob, adjacent source nodes (adjacent receiver nodes) are separated over distance $L_0 = L/(n+1)$. For clarity, Alice's node A and Bob's node B are dubbed additional source nodes C^0 and $C^{2(n+1)}$, respectively. Any source node C^{2j} has matter quantum memories, and it is connected to its adjacent receiver nodes (C^{2j-1} and C^{2j+1} for $j=1,2,\ldots,n$, C^1 for $j=0$ and C^{2n+1} for $j=n+1$) by lossy optical channels. But note that, for the application of quantum repeaters to quantum key distribution, Alice's and Bob's matter quantum memories can be regarded as virtual ideal quantum memories (see, for example, Ref. [10]). Here, in addition to the lossy optical channels, we consider the matter quantum memory to be a noisy channel, depending on the storage dimension, the memory time and the noise. For instance, if a matter quantum memory A stores a qubit during time t with dephasing, it is regarded as a phase-flip qubit channel Λ_t^A described by

$$\Lambda_t^A(\hat{\rho}) := p(t/2)\hat{I}^A\hat{\rho}\hat{I}^A + (1-p(t/2))\hat{Z}^A\hat{\rho}\hat{Z}^A, \tag{2.1}$$

where $\hat{I}^A := |0\rangle\langle 0|_A + |1\rangle\langle 1|_A$ and $\hat{Z}^A := |0\rangle\langle 0|_A - |1\rangle\langle 1|_A$ for a computational basis $\{|0\rangle_A, |1\rangle_A\}$ of system A, and

$$p(t) = \frac{1+e^{-t/\tau_{\rm c}}}{2} \tag{2.2}$$

with the coherence time $\tau_{\rm c}$. Besides, we associate the DLCZ-type quantum repeater schemes with more general linear networks by considering the minimum signalling time needed in the schemes.

Next, let us consider more specific properties of the DLCZ-type quantum repeater schemes. In this case, we usually assume that n is described by

$$n = 2^s - 1 \tag{2.3}$$

with $s = 0,1,2,\ldots$. The feature of those schemes is to use only *probabilistic* Bell measurements in *any* stage, because the schemes adopt their simple practical implementation by using linear optical elements and photon detectors [6–9]. In particular, the DLCZ-type quantum repeater protocols proceed like the following (see Supplementary Fig. 1):

(i) The scheme starts by running entanglement generation protocols between adjacent source nodes, where each source node C^{2j} sends its adjacent receiver nodes (C^{2j-1} and C^{2j+1} for $j=1,2,\dots,n$, C^1 for $j=0$ and C^{2n+1} for $j=n+1$) optical pulses *bipartitely entangled* with local matter quantum memories, through optical channels. (ii) Then, each receiver node C^{2j+1} performs *probabilistic* linear-optics-based Bell measurements on pairs of pulses arriving from its adjacent source nodes C^{2j} and C^{2j+2} ($j=0,1,\dots,n$), followed by returning the adjacent source nodes heralding signals to announce which pairs have been subjected to successful Bell measurements. (iii) Having confirmed to share entangled pairs with left-hand and right-hand source nodes separated by 2^iL_0 ($i=0,1,\dots,s-1$), source nodes perform *probabilistic* Bell measurements on halves of those pairs to obtain an extended entangled pair separated by $2^{i+1}L_0$, which is followed by sending heralding signals to announce which pairs have been subjected to successful Bell measurements. (iv) The steps (iii) are repeated until Alice and Bob obtain entangled pairs.

Let us regard this DLCZ-type quantum repeater protocol (Supplementary Fig. 1) as a linear quantum network (Supplementary Fig. 2). Since, as in step (i), source repeater node C^{2j} ($j=1,2,\dots,n$) starts by performing entanglement generation processes with the left-hand and right-hand adjacent nodes at time $t=0$ *independently*, the source repeater node C^{2j} needs to use two sets $C^{2j}_{\rm L}$ and $C^{2j}_{\rm R}$ of matter quantum memories for the left-hand and right-hand processes, respectively. This implies that node $C^{2j}_{\rm L}$ ($C^{2j}_{\rm R}$) is connected to receiver node C^{2j-1} (C^{2j+1}) by a lossy optical channel $\mathcal{O}_\eta$ parametrized by transmittance η. Besides, at time, say $t=t_{2j}$, in a step during processes (iii) and (iv), the source repeater node C^{2j} ($j=1,2,\dots,n$) applies the Bell measurements to pairs of matter quantum memories in set $C^{2j}_{\rm L}$ and in set $C^{2j}_{\rm R}$. Thus, from time $t=0$ to time $t=t_{2j}$, at least, we need to store quantum information in the matter quantum memories, implying that each of nodes $C^{2j}_{\rm L}$ and $C^{2j}_{\rm R}$ inputs quantum information into noisy quantum channel $\mathcal{M}_{t_{2j}}$ parametrized by the memory time t_{2j}. Since the Bell measurements at time $t=t_{2j}$ are considered to be applied to the outputs of the quantum channels $\mathcal{M}_{t_{2j}}$, the outputs should be held by a single node, say $C^{2j}_{\rm F}$. Hence, we can regard the sequence from the entanglement generation to the entanglement swapping as a protocol working over a linear quantum network as in Supplementary Fig. 2, where node $C^{2j}_{\rm L}$ (node $C^{2j}_{\rm R}$) is connected to node $C^{2j}_{\rm F}$ by the noisy channel $\mathcal{M}_{t_{2j}}$ as well as to node C^{2j-1} (C^{2j+1}) by the lossy optical channel $\mathcal{O}_\eta$ and all the nodes are allowed to use the quantum channels and arbitrary LOCC. Therefore, the DLCZ-type quantum repeater schemes with matter quantum memories can be considered to work over a linear network with vertices $V=\{A,C^1,C^2_{\rm L},C^2_{\rm F},C^2_{\rm R},C^3,\dots,C^{2n-1},C^{2n}_{\rm L},C^{2n}_{\rm F},C^{2n}_{\rm R},C^{2n+1},B\}$ connected by the noisy channels $\{\mathcal{M}_{t_{2j}}\}_{j=1,2,\dots,n}$ and lossy optical channels $\mathcal{O}_\eta$.

In order to derive the upper bound on the performance of the linear network by using the formula (2), we need to specify the noisy channels $\{\mathcal{M}_{t_{2j}}\}_{j=1,2,\dots,n}$ and lossy optical channels $\mathcal{O}_\eta$. Since the distance between adjacent source and receiver nodes is $L_0/2$, transmittance η of the lossy optical channels $\mathcal{O}_\eta$ is regarded as

$$\eta=\eta_{\rm c}\eta_{L_0/2}, \tag{2.4}$$

where $\eta_{\rm c}$ is the coupling efficiency and $\eta_l := e^{-l/l_{\rm att}}$ is the transmittance of the optical fibre with the length l and the attenuation length $l_{\rm att}$. In this case, the squashed entanglement of channel $\mathcal{O}_\eta$ is upper bounded [1] by

$$E_{\rm sq}(\mathcal{O}_\eta)\le 2\log_2\left(\frac{1+\eta}{1-\eta}\right)=2\log_2\left(\frac{1+\eta_{\rm c}\eta_{L_0/2}}{1-\eta_{\rm c}\eta_{L_0/2}}\right). \tag{2.5}$$

On the other hand, the memory time t_{2j} is determined by the speed v of light used for the entanglement generation and for the transmission of the heralding signals. As can be confirmed in Supplementary Fig. 1, the memory time t_{2j} depends on the locations of the source nodes. In particular, for $i=0,1,2,\dots,s-1$, there are 2^i source repeater nodes C^{2j} that need the memory time $t_{2j}=L/(2^{i+1}v)=[(n+1)L_0]/(2^{i+1}v)=(2^sL_0)/(2^{i+1}v)$. In addition, for simplicity, we assume that the quantum memory can be regarded as a phase-flip qubit channel in Eq. (2.1), that is, $\mathcal{M}_t=\Lambda_t$. Then, note that the squashed entanglement of the phase-flip channel Λ_t is upper bounded [2] by

$$E_{\rm sq}(\Lambda_t)\le h\left(\frac{1+2\sqrt{p(t/2)\,(1-p(t/2))}}{2}\right) \tag{2.6}$$

with the binary entropy function h. Therefore, the formula (2) for the average obtained *secret bits* per average total channel use is rewritten as

$$\frac{\langle\log_2 d_{\boldsymbol{k}_l}\rangle_{\boldsymbol{k}_l}}{\bar{m}_l}\le\frac{1}{2^{s+1}[E_{\rm sq}(\mathcal{O}_\eta)]^{-1}+\sum_{i=0}^{s-1}2^{i+1}[E_{\rm sq}(\Lambda_{L/(2^{i+1}v)})]^{-1}}+\frac{g(\epsilon)}{\bar{m}_l} \tag{2.7}$$

$$\le\frac{1}{2^s\left[\log_2\left(\frac{1+\eta_{\rm c}\eta_{L_0/2}}{1-\eta_{\rm c}\eta_{L_0/2}}\right)\right]^{-1}+\sum_{i=0}^{s-1}2^{i+1}\left[h\left(\frac{1+2\sqrt{p(L/(2^{i+2}v))(1-p(L/(2^{i+2}v)))}}{2}\right)\right]^{-1}}+\frac{g(\epsilon)}{\bar{m}_l}. \tag{2.8}$$

On the other hand, if the quantum repeaters are used for generating entanglement between Alice and Bob, rather than a secret key, Alice and Bob also need to have matter quantum memories to keep quantum information during time L/v. Therefore, the formula (2) for the average obtained *ebits* per average total channel use is reduced to

$$\frac{\langle \log_2 d_{\boldsymbol{k}_l} \rangle_{\boldsymbol{k}_l}}{\bar{m}_l} \leq \frac{1}{2^{s+1}[E_{\rm sq}(\mathcal{O}_\eta)]^{-1} + \sum_{i=0}^{s-1} 2^{i+1}[E_{\rm sq}(\Lambda_{L/(2^{i+1}v)})]^{-1} + 2[E_{\rm sq}(\Lambda_{L/v})]^{-1}} + \frac{g(\epsilon)}{\bar{m}_l}. \tag{2.9}$$

Equations (2.8) and (2.9) must be upper bounds on the performance of the DLCZ-type quantum repeater schemes. However, note that the upper bounds may overestimate the performance of the schemes. For instance, given only practical noisy matter quantum memories $\mathcal{M}_{t_{2j}}$, what we can do at best in the original setup (Supplementary Fig. 1) is just to combine them to have fault-tolerant quantum memories from node $C_{\rm L}^{2j}$ ($C_{\rm R}^{2j}$) to node $C_{\rm F}^{2j}$ by using a *one-way* quantum error correcting code, rather than *two-way* quantum error correcting codes or entanglement distillation. This comes from the fact that classical communication between them is limited to one-way from nodes $C_{\rm L}^{2j}$ and $C_{\rm R}^{2j}$ to node $C_{\rm F}^{2j}$ along the arrow of time in the original setup. However, our bound (1) or (2) does not reflect this restriction, because it is derived over the linear quantum network in Supplementary Fig. 2 without any restriction for classical communication. But, notably, such ultimate bounds (2.8) and (2.9) are useful to understand how much coherence time of matter quantum memories is, at least, needed to make those schemes useful. In fact, since the upper bound (2.8) [(2.9)] optimized over s for the choice of $\epsilon \to 0$, $\tau_{\rm c} = 100$ μs and $\eta_{\rm c} = 0.9$ has the same scaling as the intercity QKD protocols (with the performance in the order of $\eta_{L/2}$) [a point-to-point entanglement distribution protocol (with the performance in the order of η_L)] as in Fig. 3a [Fig. 3b], the coherence time $\tau_{\rm c}$ should be, at least, larger than hundred microseconds for enjoying the blessing of the DLCZ-type quantum repeaters even if we are allowed to use any kind of quantum error correction and entanglement distillation over the linear quantum network.

Supplementary Note 3. PROOF FOR EQ. (4)

In Eq. (1) or Supplementary Note 1, we have treated a quantum internet protocol as if it supplies only a pair of clients, called Alice A and Bob B, with secret bits or ebits. However, as noted in Discussion, our bound applies to multiple-pair cases where multiple pairs of parties try to establish secret bits or ebits at the same time, which gives a bound represented by Eq. (4). Here we present a proof for this.

Suppose that there are m pairs of clients labelled by an index j so that a node $A^j \in V$ would like to share secret bits or ebits with another node $B^j \in V$ for $j = 1, 2, \ldots, m$ by using a quantum network associated with a graph $G = (V, E)$. We also assume that there is a quantum internet protocol which presents pair $A^j B^j$ with $\log_2 d_{\boldsymbol{k}_l}^{(j)}$ secret bits or ebits within an error $\epsilon (> 0)$ with probability $p_{\boldsymbol{k}_l}$ for all $j = 1, 2, \ldots, m$. For this protocol, let us fix a subset $V' \subset V$ of vertices. For this V', we write $j \in J_{V' \leftrightarrow V \setminus V'}$ when $A^j \in V'$ and $B^j \in V \setminus V'$ hold or when $B^j \in V'$ and $A^j \in V \setminus V'$ hold.

From the definition of the quantum internet protocol, at least, clients $S_{V'} := \bigotimes_{j \in J_{V' \leftrightarrow V \setminus V'}} A^j B^j$ can distil $\log_2 d_{\boldsymbol{k}_l}^{(j)}$ bipartite secret bits or ebits ($j \in J_{V' \leftrightarrow V \setminus V'}$) within error ϵ from their final state $\hat{\rho}_{\boldsymbol{k}_l}^{S_{V'}}$ (which is the reduced density operator of the output final state $\hat{\rho}_{\boldsymbol{k}_l}^{V}$ for the whole system V). This implies

$$\left\| \hat{\rho}_{\boldsymbol{k}_l}^{S_{V'}} - \bigotimes_{j \in J_{V' \leftrightarrow V \setminus V'}} \hat{\tau}_{d_{\boldsymbol{k}_l}^{(j)}}^{A^j B^j} \right\|_1 \leq \epsilon \tag{3.1}$$

for the ideal target states $\hat{\tau}_{d_{\boldsymbol{k}_l}^{(j)}}^{A^j B^j}$, from each of which we can distil $\log_2 d_{\boldsymbol{k}_l}^{(j)}$ secret bits or ebits. Then, from Theorem 2 in Ref. [4], we have

$$\sum_{j \in J_{V' \leftrightarrow V \setminus V'}} \log_2 d_{\boldsymbol{k}_l}^{(j)} \leq E_{\rm sq}^{\mathcal{H}_{S_{V'} \cap V'}^{l|\boldsymbol{k}_{l-1}} : \mathcal{H}_{S_{V'} \cap (V \setminus V')}^{l|\boldsymbol{k}_{l-1}}} (\hat{\rho}_{\boldsymbol{k}_l}^{S_{V'}}) + g(\epsilon) \tag{3.2}$$

$$\leq E_{\rm sq}^{\mathcal{H}_{V'}^{l|\boldsymbol{k}_{l-1}} : \mathcal{H}_{V \setminus V'}^{l|\boldsymbol{k}_{l-1}}} (\hat{\rho}_{\boldsymbol{k}_l}^{V}) + g(\epsilon). \tag{3.3}$$

Through the similar discussion in Supplementary Note 1 with V_A and V_B rephrased as V' and $V \setminus V'$, respectively, we conclude Eq. (4):

$$\sum_{j \in J_{V' \leftrightarrow V \setminus V'}} \sum_{\boldsymbol{k}_l} p_{\boldsymbol{k}_l} \log_2 d_{\boldsymbol{k}_l}^{(j)} \leq \sum_{i=0}^{l-1} \sum_{\boldsymbol{k}_i \in K_{V' \leftrightarrow V \setminus V'}} p_{\boldsymbol{k}_i} E_{\rm sq}(\mathcal{N}^{e_{\boldsymbol{k}_i}}) + g(\epsilon). \tag{3.4}$$

Supplementary References

[1] Takeoka, M., Guha, S. & Wilde, M. M. Fundamental rate-loss tradeoff for optical quantum key distribution. *Nat. Commun.* **5**, 5235 (2014).

[2] Takeoka, M., Guha, S. & Wilde, M. M. The squashed entanglement of a quantum channel. *IEEE Trans. Inf. Theory* **60**(8), 4987-4998 (2014).

[3] Horodecki, K., Horodecki, M., Horodecki, P. & Oppenheim, J. Secure key from bound entanglement. *Phys. Rev. Lett.* **94**, 160502 (2005).

[4] Wilde, M. M. Squashed entanglement and approximate private states. Preprint at http://arxiv.org/abs/1606.08028 (2016).

[5] Christandl, M. & Winter, A. 'Squashed entanglement': an additive entanglement measure. *J. Math. Phys.* **45**, 829-840 (2004).

[6] Duan, L.-M., Lukin, M. D., Cirac, J. I. & Zoller, P. Long-distance quantum communication with atomic ensembles and linear optics. *Nature* **414**, 413-418 (2001).

[7] Kok, P., Williams, C. P. & Dowling, J. P. Construction of a quantum repeater with linear optics. *Phys. Rev. A* **68**, 022301 (2003).

[8] Sangouard, N., Simon, C., de Riedmatten, N. & Gisin, N. Quantum repeaters based on atomic ensembles and linear optics. *Rev. Mod. Phys.* **83**, 33-80 (2011).

[9] Azuma, K., Takeda, H., Koashi, M. & Imoto, N. Quantum repeaters and computation by a single module: Remote nondestructive parity measurement. *Phys. Rev. A* **85**, 062309 (2012).

[10] Azuma, K., Tamaki, K. & Lo, H.-K. All-photonic quantum repeaters. *Nat. Commun.* **6**, 6787 (2015).
