# Supplementary material for: Fundamental rate-loss trade-off for the quantum internet
Source: Nat Commun. 2016 Nov 25;7:13523. doi: 10.1038/ncomms13523 (PMC5133617; doi:10.1038/ncomms13523)
Supplement: Peer Review File [file ncomms13523-s2.pdf]

PEER REVIEW FILE

Reviewers' comments:

Reviewer #1 (Remarks to the Author):

A. Review of relevant prior work:

(a) Takeoka, Guha and Wilde (TGW) found a single-letter-expression upper bound for the two-way private capacity P_2 of a general quantum channel (doi:10.1038/ncomms6235) - the maximum rate at which the Alice and Bob can generate shared secret (or, equivalently privately communicate) over a quantum channel, when unlimited authenticated-yet-public two-way classical communication is available to them as an ancillary resource. The TGW upper bound is given in terms of the "squashed entanglement" of the quantum channel. TGW evaluated their rate upper bound for a pure-loss bosonic / optical channel of input-output power transmissivity \eta; 0 <= \eta <= 1, and found the upper bound: log_2[(1 + \eta)/(1 - \eta)] bits/mode. This bound approximates to 2.89 \eta bits/mode, when \eta << 1 (high loss). Evaluating the TGW bound involves a minimization of the squashed entanglement of a channel over all "squashing maps" - a local quantum operation done on the quantum system available to the Eavesdropper. In evaluating their upper bound for the lossy bosonic channel, TGW took a simple squashing map (a beamsplitter), hence their bound may not be tight. They showed however that the best known achievable rate for P_2 is given by the "reverse coherent information" of a quantum channel, which translated for the pure-loss bosonic channel yields log_2[1/(1 - \eta)] bits/mode which approximates to 1.44 \eta, when \eta << 1. So, they left at most a factor of 2 gap between the best-known achievable rate and their upper bound. One main implication of the TGW bound was that the direct-transmission QKD rate (i.e., without assistance of repeater nodes) must decay linearly with the channel's transmittance (hence exponentially with distance in fiber).

(b) Pirandola, Laurenza, Ottaviani and Banchi (PLOB) found a way to upper bound the P_2 of a quantum channel using the relative entropy of entanglement of a quantum channel. They called their bounding technique "teleportation stretching", and works only for a special class of quantum channels, which they term "stretchable channels". Luckily however, the pure loss bosonic channel happens to be a "stretchable channel", so the PLOB upper bound applies to this channel. The upper bound works out to be log_2[1/(1 - \eta)] bits/mode, which matches exactly with the best known lower bound (see above), hence established this expression to be the P_2 of the pure-loss bosonic channel. This was a big step, since the P_2 of a general quantum channel is hard to evaluate, and is not known to be additive in general.

The pure-loss bosonic channel has special relevance to optical-frequency QKD since the primary source of channel imperfection is caused due to loss. So, the PLOB bound has an advantage for this (pure-loss) channel over the TGW bound, since it closes the factor-of-2 gap in the high-loss regime, and exactly establishes the secret key agreement capacity. But, with other non-idealities added to the channel, it is not clear which of the two techniques (TGW or PLOB) provides a tighter upper bound on P_2. The TGW bound will apply to arbitrary quantum channels, and the PLOB bound will apply only if the channel is "stretchable", but many practical bosonic channels are stretchable as shown by Pirandola and co-authors.

B. The context of the current work of NCOMMS-16-04048-T, and related work:

Problem P - Quantum repeaters are devices which when inserted along the length of an optical channel can help generate shared secret at a rate that surpasses the P_2 (which for the lossy bosonic

channel is the PLOB bound). Quantum repeaters need not be trusted or physically secured in order to ensure the security of the keys generated. If n quantum repeaters are inserted along the length of the channel connecting the communicating parties Alice and Bob, and if there are absolutely no physical constraints placed on the repeater nodes (i.e., the repeaters are assumed to be lossless, error-corrected, general purpose quantum computers), what is the maximum key rate achievable by Alice and Bob assuming the transmissivities of the channel segments are \eta_1, \eta_2, ..., \eta_{n+1}, with \eta_1...\eta_{n+1} = \eta being the total end-to-end transmissivity?

(a) The authors of NCOMMS-16-04048-T address the above problem P, via a very simple - yet elegant - argument, which argues that the TGW bound applied to the "worst" link in the path (the smallest eta_i) is an upper bound to the overall rate. The argument is simple - they bipartition the line lattice into two subgraphs separated by just one of the links, and consider all the nodes in the first bipartition to be possessed by Alice, and all the nodes in the first bipartition to be possessed by Bob. This clubbing of nodes clearly can only make the P_2 better. Hence the TGW bound applied on that one link connecting the two node sets in the bipartition is also an upper bound to the actual Alice-to-Bob rate. This argument is easily extended to Alice and Bob being two nodes in a quantum repeater network of a general topology with perfect repeaters, where the rate-upper bound on the Alice-to-Bob rate is the TGW bound applied to the lossiest link in a path connecting Alice and Bob, followed by taking a minimum over this upper bound for all paths from Alice to Bob. The paper makes this rigorous by extending the local operations and public communications (LOPC) considered by TGW to the above setting.

(b) Pirandola also recently considered the problem P (http://arxiv.org/pdf/1601.00966v1.pdf), and using an argument similar to the PLOB paper (see A.(b) above), he showed that the PLOB bound applied to the lossiest link in a path connecting Alice and Bob is an upper bound to the secret key rate, and one can they follow up by taking a minimum over this upper bound for all paths from Alice to Bob. He also computes an achievable lower bound, and shows that log_2[1/(1 - \eta)] bits/mode is the secret-key agreement capacity for Alice and Bob trying to generate shared secret in a quantum repeater network, where eta is the maximum over all Alice-to-Bob paths, of the transmissivity of the lossiest link in an Alice-to-Bob path.

In order to address problem P, the authors of NCOMMS-16-04048-T used the TGW technique, and Pirandola (author of http://arxiv.org/pdf/1601.00966v1.pdf) used the PLOB technique. It is but natural that the authors of NCOMMS-16-04048-T inherited the good and the bad of the TGW, and Pirandola inherited the good and the bad of the PLOB, as explained above. In particular, Pirandola's result is tighter than the bound in NCOMMS-16-04048-T if all the links in the network are pure-loss channels, but the result of NCOMMS-16-04048-T has wider applicability since the TGW bound based on squashed entanglement can be applied to the case when the links of the network are arbitrary quantum channels.

I feel that both of the above papers - NCOMMS-16-04048-T andhttp://arxiv.org/pdf/1601.00966v1.pdf combined - could make for a good article and one that might be considered for Nature Communications if appropriately messaged. I hesitate to recommend to publish NCOMMS-16-04048-T in Nature Communications in vacuum since we know that applied to the network links being purely lossy, the result of (http://arxiv.org/pdf/1601.00966v1.pdf) is superior. The results of both papers are very intuitive. That does not take away from the elegance of the techniques each paper used, but I am slightly wary of the impact of both of these papers also for the following reason:

I feel that a much more important question to ask in the context of rate limits for a quantum internet are when the repeater nodes are resource constrained. The result above states that with n

unbounded-capacity fully-error-corrected quantum repeaters inserted within a channel of transmissivity \eta, the maximum key rate achievable is -\log_2(1-\eta^{1/(n+1)}) bits/mode, where \eta^{1/(n+1)} is the transmissivity of each intermediate link with the n nodes equip-spaced. As n \to \infty, the rate goes to infinity. However, assuming repeaters to be lossless error-corrected quantum computers is not practical. A more practically relevant question to ask is if the repeater nodes have finite resources with lossy and imperfect components (where `resources' may be different physical entities depending upon the type of quantum repeater and the protocol employed), then what rate can Alice and Bob achieve, and more importantly what would it take to build repeater nodes so as to be able to significantly outperform the no-repeater direct-transmission rate bound of -\log_2(1-\eta) bits/mode.

Reviewer #2 (Remarks to the Author):

The authors derive rate bounds for quantum repeaters and similar protocols based on the recently derived bound by Takeoka, Guha and Wilde for direct quantum communication. Their approach is simple, and I see no reason to doubt the correctness of their new bounds, although other people will be in a better position to judge that aspect. However, the relevance of their bounds relies quite strongly on the authors' claim that their bound is saturated by a simple (if somewhat idealized) quantum repeater protocol. I am not convinced by the authors' estimate of the rate for that simple protocol. They seem to argue that the waiting time for n+1 independent links is given by n+1 times the waiting time for a single link. This seems to contradict the treatment in Appendix A.2 of their reference 15 (the RMP paper by Sangouard et al). I would like them to clarify this point. If I am correct, the gap between their bound and their proposed repeater protocol could be significantly wider than they think, which would impact my judgment of the relevance of these new bounds.

Dear Referees 1 and 2,

We thank both referees for your helpful comments. We have now substantially revised our manuscript to address those comments.

It is our hope that the referees will now agree that with this revision, our paper is even better than the first version and is more informative for readers.

A summary of our revision is as follows:

1) We now clarify the difference between the TGW bound [14] and the PLOB bound [16] in the introduction.

2) We now clarify the difference between our bound and Pirandola's contemporary bound [36].

3) In the previous version of our formulation for our bound (1), the total number of channel uses was assumed to be constant, and it was thus equivalent to the number l of rounds of the given internet protocol. However, we now note that, in general, the total number of channel uses can be a random variable. Hence, we have now modified the proof for our bound (1) such that it is applicable even to cases where the total number of channel uses is a random variable. As a result, for instance, the bound (4) is written in terms of the average total number $\bar{m}_l$ of channel uses, rather than the number l of rounds of the protocol.

4) We have derived the ultimate upper bound (described by Fig. 3) for the DLCZ-type quantum repeater protocols with time-dependent decay of matter quantum memories, from our main result (1). This is a non-trivial application of our method.

5) Discussion has been substantially revised to show the applicability of our bound to any multi-pair bipartite quantum communication as well as to update the progress in our field. Our discussion extends the generality of our results.

6) We have revised the manuscript to comply with the format requirements of Nature Communications. For instance, we have revised the abstract.

Dear Referee 1,

We sincerely thank Referee 1 for reviewing our manuscript and giving us a very detailed report. Your constructive comments (coloured blue in what follows) lead to a significant improvement of our result as well as our paper, as can be confirmed below and by the revised manuscript. In what follows, we would like to reply to your comments in order. Note that the reference numbers appearing below represent ones in the revised manuscript.

<u>(A) On the difference between the TGW bound and the PLOB bound:</u>

> *'A. Review of relevant prior work:*
>
> *(a) Takeoka, Guha and Wilde (TGW) found a single-letter-expression upper bound for the two-way private capacity P_2 of a general quantum channel (doi:10.1038/ncomms6235) - the maximum rate at which the Alice and Bob can generate shared secret (or, equivalently privately communicate) over a quantum channel, when unlimited authenticated-yet-public two-way classical communication is available to them as an ancillary resource. The TGW upper bound is given in terms of the "squashed entanglement" of the quantum channel. TGW evaluated their rate upper bound for a pure-loss bosonic / optical channel of input-output power transmissivity \eta; 0 <= \eta <= 1, and found the upper bound: log_2[(1 + \eta)/(1 - \eta)] bits/mode. This bound approximates to 2.89 \eta bits/mode, when \eta << 1 (high loss). Evaluating the TGW bound involves a minimization of the squashed entanglement of a channel over all "squashing maps" - a local quantum operation done on the quantum system available to the eavesdropper. In evaluating their upper bound for the lossy bosonic channel, TGW took a simple squashing map (a beamsplitter), hence their bound may not be tight. They showed however that the best known achievable rate for P_2 is given by the "reverse coherent information" of a quantum channel, which translated for the pure-loss bosonic channel yields log_2[1/(1 - \eta)] bits/mode which approximates to 1.44 \eta, when \eta << 1. So, they left at most a factor of 2 gap between the best-known achievable rate and their upper bound. One main implication of the TGW bound was that the direct-transmission QKD rate (i.e., without assistance of repeater nodes) must decay linearly with the channel's transmittance (hence exponentially with distance in fiber).*
>
> *(b) Pirandola, Laurenza, Ottaviani and Banchi (PLOB) found a way to upper bound the P_2 of a quantum channel using the relative entropy of entanglement of a quantum channel. They called their bounding technique "teleportation stretching", and works only for a special class of quantum channels, which they term "stretchable channels". Luckily however, the pure loss bosonic channel happens to be a "stretchable channel", so the PLOB upper bound applies to this channel. The upper bound works out to be log_2[1/(1 - \eta)] bits/mode, which matches exactly with the best known lower bound (see above), hence established this expression to be the P_2 of*

the pure-loss bosonic channel. This was a big step, since the P_2 of a general quantum channel is hard to evaluate, and is not known to be additive in general.

The pure-loss bosonic channel has special relevance to optical-frequency QKD since the primary source of channel imperfection is caused due to loss. So, the PLOB bound has an advantage for this (pure-loss) channel over the TGW bound, since it closes the factor-of-2 gap in the high-loss regime, and exactly establishes the secret key agreement capacity. But, with other non-idealities added to the channel, it is not clear which of the two techniques (TGW or PLOB) provides a tighter upper bound on P_2. The TGW bound will apply to arbitrary quantum channels, and the PLOB bound will apply only if the channel is "stretchable", but many practical bosonic channels are stretchable as shown by Pirandola and co-authors.'

Thank you very much for contextualizing the TGW bound and the PLOB bound precisely. We have now revised our manuscript to give a clearer context of our research. In particular, we explain the TGW bound and the PLOB bound and their advantages/disadvantages in the second paragraph of the introduction. In addition, by referring to Ref. 52 in the last sentence of the paper, we mention about the possibility of getting a better upper bound for the squashed entanglement of the lossy channel than 'log_2[(1 + \eta)/(1 - \eta)] bits/mode'. This is to show our agreement with Referee 1's comment that the upper bound may not be tight. Please take a look.

<u>(B) On the difference between our bound and Pirandola's bound:</u>

'(a) The authors of NCOMMS-16-04048-T address the above problem P, via a very simple - yet elegant - argument, which argues that the TGW bound applied to the "worst" link in the path (the smallest eta_i) is an upper bound to the overall rate. The argument is simple - they bipartition the line lattice into two subgraphs separated by just one of the links, and consider all the nodes in the first bipartition to be possessed by Alice, and all the nodes in the first bipartition to be possessed by Bob. This clubbing of nodes clearly can only make the P_2 better. Hence the TGW bound applied on that one link connecting the two node sets in the bipartition is also an upper bound to the actual Alice-to-Bob rate. This argument is easily extended to Alice and Bob being two nodes in a quantum repeater network of a general topology with perfect repeaters, where the rate-upper bound on the Alice-to-Bob rate is the TGW bound applied to the lossiest link in a path connecting Alice and Bob, followed by taking a minimum over this upper bound for all paths from Alice to Bob. The paper makes this rigorous by extending the local operations and public communications (LOPC) considered by TGW to the above setting.

(b) Pirandola also recently considered the problem P (http://arxiv.org/pdf/1601.00966v1.pdf), and using an argument similar to the PLOB paper (see A.(b) above), he showed that the PLOB bound applied to the lossiest link in a path connecting Alice and Bob is an upper bound to the secret key rate, and one can they follow up by taking a minimum over this upper bound for all paths from Alice to Bob. He also computes an achievable lower bound, and shows that log_2[1/(1 - \eta)] bits/mode is the secret-key agreement capacity for Alice and Bob trying to generate

shared secret in a quantum repeater network, where eta is the maximum over all Alice-to-Bob paths, of the transmissivity of the lossiest link in an Alice-to-Bob path.

In order to address problem P, the authors of NCOMMS-16-04048-T used the TGW technique, and Pirandola (author of http://arxiv.org/pdf/1601.00966v1.pdf) used the PLOB technique. It is but natural that the authors of NCOMMS-16-04048-T inherited the good and the bad of the TGW, and Pirandola inherited the good and the bad of the PLOB, as explained above. In particular, Pirandola's result is tighter than the bound in NCOMMS-16-04048-T if all the links in the network are pure-loss channels, but the result of NCOMMS-16-04048-T has wider applicability since the TGW bound based on squashed entanglement can be applied to the case when the links of the network are arbitrary quantum channels.'

Thank you for stating that our argument is very simple but elegant. In addition, we acknowledge Referee 1 for summarizing advantages/disadvantages of our bound and Pirandola's contemporary work [36]. According to this, we have revised our manuscript so as to clarify the difference between our bound and Pirandola's bound [36]. For instance, we now explicitly state that

'As this upper bound for point-to-point links, we simply use the TGW bound with respecting the generality of the TGW bound, in contrast to Pirandola's contemporary work[36] that instead uses the PLOB bound to obtain a good bound for multipath networks composed of optical lossy channels'

and

'Moreover, thanks to inheriting the generality of the TGW bound, in contrast to Pirandola's bounds[36] applied only to teleportation stretchable quantum channel networks, our bounds can be computed and applied to any situation that can be regarded as the quantum internet as Kimble has considered[1], including the simulation of the quantum many-body systems as well as purely quantum communication tasks'

on page 5,

'Again, note that our bound (1) is applicable to any network topology as well as arbitrary quantum channels connecting nodes, in contrast to Pirandola's one[36] with the assumption of the teleportation stretchability for quantum channels'

in the first paragraph on page 8, and

'For example, our bounds (1) and (6) would be useful for deriving the ultimate performance of the distributed quantum computation[29, 48–51] and of more practical quantum repeaters with more complicated noise models. This versatility of our bounds would be in contrast to Pirandola's bound[36] restricted to teleportation stretchable quantum channel networks. This is because one would not be surprised if a practical QIP scheme involves quantum channels without teleportation stretchability.'

in the last of the first pargarph on page 20. In addition, we mention about the difference in the formulation between our work and Pirandola's work [36], by stating

'Also, note that the left-hand side quantity—which is the average obtained secret bits or ebits per average total channel use—is different from Pirandola's measure[36] for the performance based on the restriction of $\overline{m}_l^{C^0\leftrightarrow C^1} = \overline{m}_l^{C^1\leftrightarrow C^2} = \cdots = \overline{m}_l^{C^n\leftrightarrow C^{n+1}}$*,*

in the last sentence on page 9. In particular, Pirandola considers the performance of the teleportation stretchable quantum channel networks with an assumption that the numbers of times each channel is used are the same, while our bound does not have such an assumption.

<u>(C) On Referee 1's opinion:</u>

'I feel that both of the above papers - NCOMMS-16-04048-T and http://arxiv.org/pdf/1601.00966v1.pdf combined - could make for a good article and one that might be considered for Nature Communications if appropriately messaged. I hesitate to recommend to publish NCOMMS-16-04048-T in Nature Communications in vacuum since we know that applied to the network links being purely lossy, the result of (http://arxiv.org/pdf/1601.00966v1.pdf) is superior. The results of both papers are very intuitive. That does not take away from the elegance of the techniques each paper used,'

Thank you again for stating that both of our result and Pirandola's work [36] combined might be considered for Nature Communications if appropriately messaged. Thanks to your comments, as having already been mentioned in (A) and (B), we have revised our paper to better contextualize our result and Pirandola's work [36], from which readers can easily identify advantages/disadvantages of those papers and they can intuitively understand the potential of quantum networks.

<u>(D) On Referee 1's concern:</u>

'but I am slightly wary of the impact of both of these papers also for the following reason:

I feel that a much more important question to ask in the context of rate limits for a quantum internet are when the repeater nodes are resource constrained. The result above states that with n unbounded-capacity fully-error-corrected quantum repeaters inserted within a channel of transmissivity \eta, the maximum key rate achievable is -\log_2(1-\eta^{1/(n+1)}) bits/mode, where \eta^{1/(n+1)} is the transmissivity of each intermediate link with the n nodes equip-spaced. As n \to \infty, the rate goes to infinity. However, assuming repeaters to be lossless error-corrected quantum computers is not practical. A more practically relevant question to ask is if the repeater nodes have finite resources with lossy and imperfect components (where `resources' may be different physical entities depending upon the type of quantum repeater and the protocol employed), then what rate can Alice and Bob achieve, and more importantly what would it take to build repeater nodes so as to be able to significantly outperform the no-repeater direct-transmission rate bound of -\log_2(1-\eta) bits/mode.'

Thank you for giving us this concern on the applicability of our bound to more practical quantum repeaters. In order to address this concern, we have substantially revised our manuscript to show how to apply our bound (1) to a representative class of practical quantum repeaters, called DLCZ-type quantum repeater protocols. In this case, as Referee 1 states, we are required to include imperfect components other than the optical lossy channels, that is, matter quantum memories with time-dependent decay. Moreover, as Referee 1 expects, our bound clarifies the following nontrivial necessary condition that the DLCZ-type quantum repeater schemes can outperform non-quantum-repeater schemes: The coherence time of matter quantum memories should be, at least, 100 μs (unless repeated quantum error correction steps are applied to quantum memories while waiting for heralding signals). Of course, this is merely an example of many potential applications of our bound to practical quantum repeater schemes. But, we believe that the newly added example is significant enough to trigger further considerations for the potential applications to the practical schemes. Therefore, we hope that the added application of our bound results in dispelling Referee 1's concern.

In particular, to include this new result, we first derive an upper bound (4) applied to the general linear network, from our bound (1). Then, by introducing a correspondence between the DLCZ-type quantum repeater protocols and a linear network composed of optical lossy channels and noisy qubit channels, we present upper bounds (2.8) and (2.9) in Supplementary Note 2 on the performance of the DLCZ-type quantum repeater protocols, from the bound (4). These upper bounds are estimated as in Fig. 3. From Fig. 3, the necessary minimum coherence time, 100 μs, can easily be confirmed. In addition, in the calculation for Fig. 3, since we include the effect of the coupling loss of photons to the optical fibres, the number of repeater nodes is determined to maximize the upper bounds (2.8) and (2.9), which is different from the situation, '*As n \to \infty, the rate goes to infinity*'.

Furthermore, on p. 18 and 19 of our revised manuscript, we now show how our bound could be applied to the multiple pair case where multiple pairs of parties would like to establish secure keys or ebits with each other. This is a non-trivial extension of our result.

Again, we would like to thank Referee 1 for recommending us to contextualize our work more precisely and for triggering us to consider the applicability of our bound (1) to practical quantum repeaters and to extend our result to the multiple pair case. The revised manuscript is much more informative for readers than the first version and it clearly shows the versatility of our bound (1). Thank you very much for presenting us with very constructive comments.

Dear Referee 2,

Thank you for reviewing our paper and for admitting the correctness of our new bounds. In addition, thank you very much for giving us your concern. In what follows, we would like to reply to your comments (coloured blue in what follows) in order. Note that the reference numbers appearing below represent ones in the revised manuscript.

(a) On the correctness on our bounds:

> *'The authors derive rate bounds for quantum repeaters and similar protocols based on the recently derived bound by Takeoka, Guha and Wilde for direct quantum communication. Their approach is simple, and I see no reason to doubt the correctness of their new bounds, although other people will be in a better position to judge that aspect. '*

Thank you for admitting the correctness of our new bounds.

(b) On Referee 2's concern:

> *'However, the relevance of their bounds relies quite strongly on the authors' claim that their bound is saturated by a simple (if somewhat idealized) quantum repeater protocol. I am not convinced by the authors' estimate of the rate for that simple protocol. They seem to argue that the waiting time for n+1 independent links is given by n+1 times the waiting time for a single link. This seems to contradict the treatment in Appendix A.2 of their reference 15 (the RMP paper by Sangouard et al). I would like them to clarify this point. If I am correct, the gap between their bound and their proposed repeater protocol could be significantly wider than they think, which would impact my judgment of the relevance of these new bounds.'*

Note that our bound and the performance for our 'idealized qubit-based protocol' are written in terms of how many times quantum channels are used, rather than the waiting time appearing in Appendix A.2 of the RMP by Sangouard *et al*. [17]. Therefore, the performance measures are merely different between our paper and Ref. [17], and our estimation of the performance for our 'idealized qubit-based protocol' is correct. In other words, our paper *does not* use the waiting time as the performance measure for the protocols, and thus, we *do not* argue that '*the waiting time for n+1 independent links is given by n+1 times the waiting time for a single link'* for our idealized qubit-based protocol. We just conclude that there is no scaling gap between our idealized qubit-based quantum repeater protocol and our bound (5) in terms of the *same* performance measure, that is, the average obtained secret bits or ebits per average total channel use.

In our paper, we stick to using the number of channel uses, rather than the waiting time, to keep the generality of our formulation. In general, in order to translate the quantity written with the number of channel uses into that with the waiting time, we need to have a 'frequency' representing how many times of quantum channels can be used per time. Hence, once given such a frequency, our bounds—written in terms of the number of channel uses—should be rephrased in terms of the waiting time like the performance measure in Ref. [17]. However, in fact, the frequency depends on many factors in the realization, such as initialization time of devices, operation time, clock frequencies of devices, signalling time of heralding signals, availability of multiplexing and so on. In other words, the frequency is not an invariable quantity, and it could increase with the development of physical devices used for the realization. Therefore, it is more fundamental to evaluate the performance in terms of the number of channel uses, rather than the waiting time as in Ref. [17]. It is merely the fact that Ref. [17] can adopt the waiting time for the performance measure, because the goal of Ref. [17] is to compare similar protocols based on similar physical devices.

Another thing to note is that, for a linear chain with n channels (i.e., there are $(n+1)$ nodes and Alice and Bob are the two end nodes), to pass a qubit from Alice to Bob requires already a minimal usage of n channels. So, there is a factor of n that one needs to take into account in the counting of the number of channel uses per generated secret bit or ebit.

Again, we would like to thank Referee 2 for admitting the correctness of our bounds and for giving us a chance to reconsider the difference in the performance measure between our paper and Ref. [17]. Through this reviewing process, our paper has been substantially revised to be much more informative for readers than the first version. In addition, we have added a discussion about the ultimate performance of the DLCZ-type quantum repeaters [17,22,23,29] to our revised manuscript. Furthermore, on p. 18 and 19 of our revised manuscript, we now show how our bound could be applied to the multiple pair case where multiple pairs of parties would like to establish secure keys or ebits with each other. This is a non-trivial extension of our result. The above two extensions of our results show the versatility of our bound (1). Therefore, we would be very happy if Referee 2 finds this revised manuscript scientifically more interesting.

REVIEWERS' COMMENTS:

Reviewer #1 (Remarks to the Author):

I am satisfied with the careful modifications the authors have made to their manuscript. I also really like the addition of the rate upper bound for the DLCZ scheme with time-dependent memory decay. I would recommend publication of the paper, after the following comment is addressed:

From the caption of Fig. 3, it is not clear to me what is n, the number of DLCZ repeater nodes. Are you taking an envelope over all n? It would be nice to state that. In fact, for clarity, it might be worth it to show (perhaps just for one of the \tau_c values) the rate-vs.-distance plots for a few different values of n, i.e., with n held fixed, and show the rate-distance envelope over all n. This will make the point clear that increasing n always does not help, unlike what the unrestricted UB predicts, and that at any given range L, there is an optimum value of the number of DLCZ repeater nodes built with lossy memories.

Reviewer #2 provided confidential remarks to the editor, explaining that they find reason in your response, but they remain unconvinced of the practical relevance of your bounds.

Dear Referee 1,

Thank you very much for reviewing our paper again and giving us constructive comments. Attached please find the PDF files showing all the corrections in this round. In what follows, we reply to your comments (colored blue in what follows) in order.

> *I am satisfied with the careful modifications the authors have made to their manuscript.*

Note that these modifications had been triggered by the referee report in the first round. We are also satisfied with our current manuscript. In this regard, we really thank you for giving us constructive comments throughout the review process.

> *I also really like the addition of the rate upper bound for the DLCZ scheme with time-dependent memory decay. I would recommend publication of the paper, after the following comment is addressed:*

Thank you for stating that you really like the upper bound on the rate for the DLCZ scheme with time-dependent memory decay. We also thank you for recommending the publication of our paper.

> *From the caption of Fig. 3, it is not clear to me what is n, the number of DLCZ repeater nodes. Are you taking an envelope over all n? It would be nice to state that. In fact, for clarity, it might be worth it to show (perhaps just for one of the \tau_c values) the rate-vs.-distance plots for a few different values of n, i.e., with n held fixed, and show the rate-distance envelope over all n. This will make the point clear that increasing n always does not help, unlike what the unrestricted UB predicts, and that at any given range L, there is an optimum value of the number of DLCZ repeater nodes built with lossy memories.*

As you infer, n is a parameter associated with the number of repeater nodes, and Fig. 3 shows only envelopes of the upper bounds over all n's. For simplicity, instead of showing upper bounds for a few different values of n's, we have decided to stress that Fig. 3 shows the envelopes.

In particular, we now define n clearly through

> *Conventionally, these schemes use the set of repeater nodes* $\{C^j\}_{j=1,2,\ldots,2n+1}$ *—which is composed of source repeater nodes* $\{C^{2j}\}_{j=1,2,\ldots,n}$ *and receiver repeater nodes* $\{C^{2j+1}\}_{j=0,1,\ldots,n}$ *—between Alice* $A(=:C^0)$ *and Bob* $B(=:C^{2n+2})$*, where* $n = 2^s - 1$ *for* $s = \{0,1,2,\ldots\}$.

in the second paragraph on page 15 and

> *Conventionally, the protocols use repeater nodes* $\{C^j\}_{j=1,2,\ldots,2n+1}$ *between Alice and Bob, which are located at regular intervals and connected by lossy optical channels with each other, as in Fig. 1(b).*

in the caption of Fig. 3. Then, we explicitly state

> *Under these conditions, in Fig. 3, the number n associated with the number of repeater nodes is optimized to maximize the upper bounds. The existence of optimal n here—which is in contrast to the case for upper bounds for purely optical channel networks as in Fig. 2—stems from the existence of local errors/loss in the repeater nodes.*

in the first paragraph on page 17, and

> *The existence of optimal n here---which is in contrast to the case for upper bounds in Fig.~2---stems from the existence of local errors/loss in the repeater nodes.*

in the caption of Fig. 3. Thank you so much for suggesting us to refine the presentation of the results in Fig. 3.

In summary, we thank you very much for your helpful comments. We sincerely hope that you will now find our revised manuscript suitable for immediate publication in *Nature Communications.*

Yours Sincerely,
Koji Azuma, Akihiro Mizutani, and Hoi-Kwong Lo
